# Interplay Between Glucose Metabolism and Chromatin Modifications in Cancer

**DOI:** 10.3389/fcell.2021.654337

**Published:** 2021-04-27

**Authors:** Rui Ma, Yinsheng Wu, Shanshan Li, Xilan Yu

**Affiliations:** ^1^State Key Laboratory of Biocatalysis and Enzyme Engineering, Environmental Microbial Technology Center of Hubei, School of Life Sciences, Hubei University, Wuhan, China; ^2^College of Biomedicine and Health, Huazhong Agricultural University, Wuhan, China

**Keywords:** metabolism, epigenetic modifications, gene transcription, tumorigenesis, histone modifications

## Abstract

Cancer cells reprogram glucose metabolism to meet their malignant proliferation needs and survival under a variety of stress conditions. The prominent metabolic reprogram is aerobic glycolysis, which can help cells accumulate precursors for biosynthesis of macromolecules. In addition to glycolysis, recent studies show that gluconeogenesis and TCA cycle play important roles in tumorigenesis. Here, we provide a comprehensive review about the role of glycolysis, gluconeogenesis, and TCA cycle in tumorigenesis with an emphasis on revealing the novel functions of the relevant enzymes and metabolites. These functions include regulation of cell metabolism, gene expression, cell apoptosis and autophagy. We also summarize the effect of glucose metabolism on chromatin modifications and how this relationship leads to cancer development. Understanding the link between cancer cell metabolism and chromatin modifications will help develop more effective cancer treatments.

## Introduction

Tumor cells need to change their metabolism to support their demands for rapid growth and proliferation, so called metabolism reprogram (Pavlova and Thompson, 2016). This metabolic reprogram enables cells to synthesize a large amount of precursors for biomacromolecule synthesis (Pavlova and Thompson, 2016). The extensive studied metabolic reprogram is aerobic glycolysis, also known as the “Warburg effect.” That is, cancer cells preferentially convert pyruvate, the end product of glycolysis into lactate instead of transporting pyruvate into the mitochondria for oxidative phosphorylation. Although aerobic glycolysis is a less efficient way to produce energy (2 ATP/glucose), it helps accumulate a large amount of metabolite precursors for biosynthesis of macromolecules, i.e., nucleic acids, fatty acids, and amino acids (Hanahan and Weinberg, 2011). Initially, the mitochondria in tumor cells was thought to have defects, which makes them unable to perform oxidative phosphorylation and highly dependent on glycolysis (Dang and Semenza, 1999). However, the function of mitochondria in most tumor cells is intact. Recent studies show that tumor cells mainly use the tricarboxylic acid (TCA) cycle in the G1 phase and prefer glycolysis in the S phase (Liu et al., 2021), suggesting that both TCA cycle and glycolysis are important for tumor cells.

Many metabolic enzymes and metabolites have non-metabolic functions in tumorigenesis, including regulation of chromatin modifications, gene transcription, DNA damage, etc. (Yu and Li, 2017). These non-metabolic functions provide useful clues to develop more efficient anti-cancer therapy. In this review, we described the functions of metabolic enzymes and metabolites from glycolysis, gluconeogenesis, and TCA cycle in tumorigenesis with an emphasis on their non-metabolic functions. We also described how they are regulated and their effects on chromatin modifications.

## Metabolic Regulation of Tumor Cell Proliferation

### The Role of Glycolytic Enzymes in Tumorigenesis

#### Hexokinase

**Figure 1:**
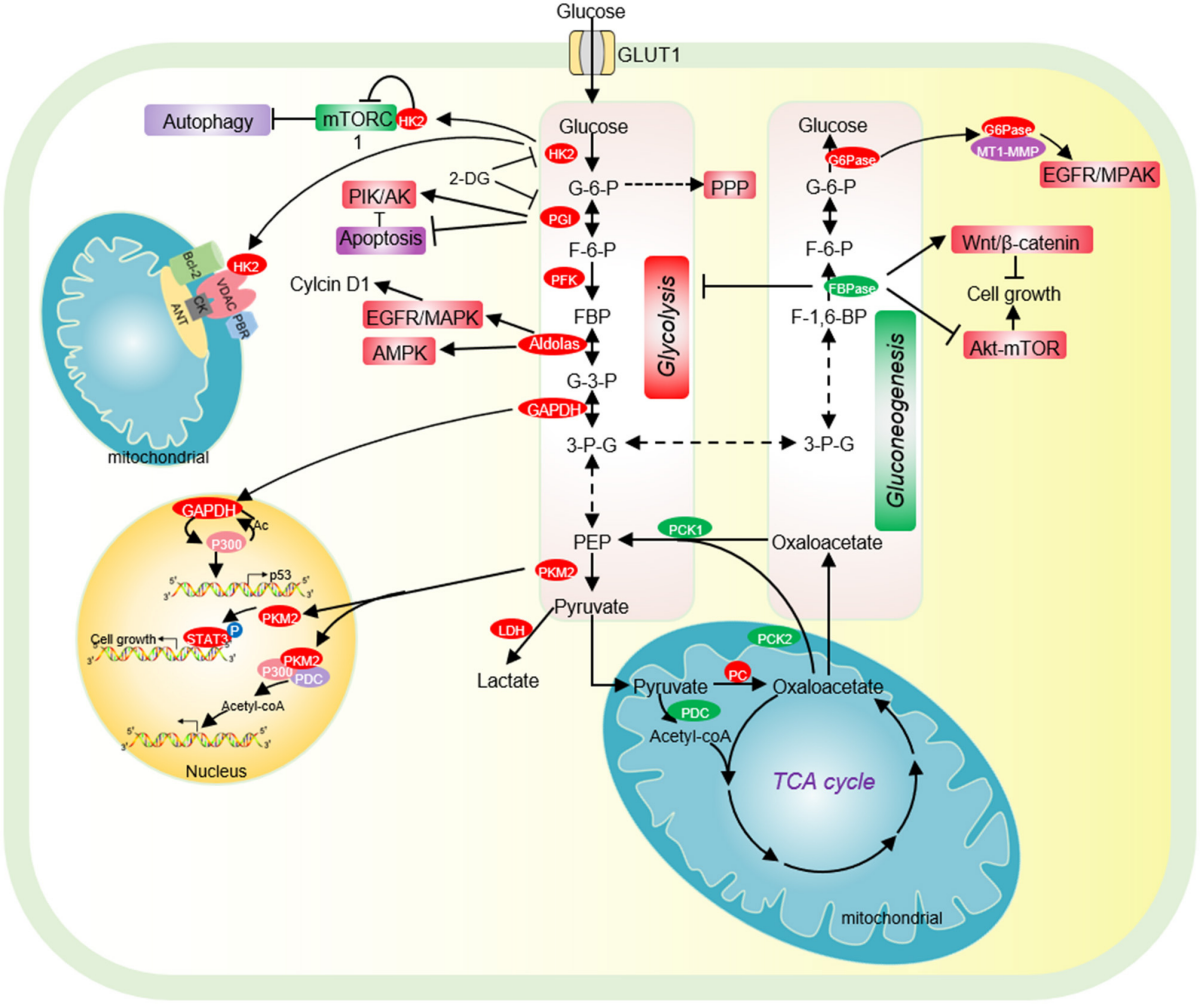
The effect of glycolysis and gluconeogenesis on tumor cell proliferation. Cancer cells have increased glucose uptake, leading to accelerated glycolysis and biomass accumulation. The high expression of GLUT1, HK2, PGI, PFK, Aldolase, GAPDH, and PKM2 significantly accelerates aerobic glycolysis. Increased lactate dehydrogenase (LDH) activity and decreased pyruvate dehydrogenase (PDC) activity result in increased lactate export, attenuated TCA cycle and diversion of glycolysis to pentose phosphate pathway (PPP). HK2 not only directly binds to mTORC1 to activate autophagy, but also interacts with VDAC to protect tumor cells from apoptosis. PGI inhibits cell apoptosis by inhibiting the expression of Apaf-1 and caspase-9 and activating the PI3K/Akt signaling pathway. Acetylated nuclear GAPDH promotes tumorigenesis by activating p300 and promoting p53 expression. Aldolase promotes cell cycle progression through the EGFR/MAPK signaling pathway, and activates AMPK to maintain tumor cell survival in glucose deficiency. Nuclear PKM2 performs a variety of non-metabolic functions to promote cell cycle progression. Low expression of pyruvate carboxylase (PC), the key enzyme of gluconeogenesis, is essential for the proliferation of some cancer cells. PC is essential for the synthesis of fatty acid and glycerol in cancer cells. The low expression of the key enzyme PEPCK of gluconeogenesis is beneficial for the proliferation of cancer cells. FBPase acts as a tumor suppressor and inhibits the proliferation of tumor cells. G6PT promotes the invasion of glioblastoma by interacting with MT1-MMP. HK2, hexokinase 2; G-6-P, glucose-6-phosphate; PGI, phosphoglucose isomerase; F-6-P, fructose-6-phosphate; FBP, fructose-1,6-biphosphate; G-3-P, glyceraldehyde-3-phosphate; 3-P-G, 3-phosphoglycerate; GAPDH, Glyceraldehyde-3-phosphate dehydrogenase; PKM2, Pyruvate kinase M2; LDH, lactate dehydrogenase; PC, Pyruvate carboxylase; PCK1/2, phosphoenolpyruvate carboxykinase 1/2; PDC, pyruvate dehydrogenase complex; PEP, phosphoenolpyruvate; PKM2, pyruvate kinase M2; FBPase, fructose-1,6-bisphosphatase; G6Pase, glucose-6-phosphatase.

Hexokinase (HK2) is the first rate-limiting enzyme in glycolysis, which is highly expressed in tumor cells and acts as a potential target for cancer treatment (Chen J. et al., 2014). HK2 binds to the mitochondrial membrane *via* its interaction with the outer membrane porin protein, voltage-dependent anion channel (VDAC) (Figure 1). VDAC is a critical channel that regulates the release rate of mitochondrial intermembrane pro-apoptotic proteins, such as cytochrome *c* (Linden et al., 1982). The interaction between HK2 and VDAC inhibits the release of intermembrane pro-apoptotic proteins, thereby protecting tumor cells from apoptosis (Figure 1) (Linden et al., 1982). HK2 has also been reported to interact with the mammalian target of rapamycin complex 1 (mTORC1) by binding to its subunit, regulatory-associated protein of mTOR (Raptor) (Figure 1) (Roberts et al., 2014). Upon glucose deprivation, HK2 directly binds to mTORC1 to inhibit its function and activate the protective autophagy pathway (Roberts et al., 2014).

The expression of HK2 is closely correlated to the occurrence of cancers such as laryngeal cancer and breast cancer, metastasis of breast cancer cells and poor prognosis of patients (Palmieri et al., 2009; Kwee et al., 2012; Min et al., 2013). HK2 expression is regulated by microRNAs, long non-coding RNA and various transcript factors. The expression of miR-143 is negatively correlated with the HK2 expression level in a variety of cancers, including prostate cancer, breast cancer, and head and neck squamous cell carcinoma (HNSCC) (Zhang et al., 2017). miR-143 directly targets the 3′UTR (3′ untranslated region) of HK2 to repress its expression (Peschiaroli et al., 2013). miR-155 down-regulates the transcription of miR-143 by targeting its transcription activator C/EBP β, which then affects HK2 expression (Jiang et al., 2012). The long non-coding RNA UCA1 up-regulates HK2 transcription by repressing miR-203 and upregulated UCA1 enhances the malignant phenotype of esophageal cancer (Liu et al., 2020). HK2 is also regulated by transcription factors. For example, hypoxia-inducible factor 1α (HIF-1α) binds the promoter of HK2 to activate HK2 transcription, which accelerates glycolysis and facilitates cutaneous squamous cell carcinoma (cSCC) development (Zhou et al., 2018). In addition, the expression of HK2 is activated by transforming growth factor-β (TGF-β) and this activation requires transcription factors like Smad2, Smad3, and c-Myc (Yin et al., 2019).

#### Phosphoglucose Isomerase

Phosphoglucose isomerase (PGI) is the second glycolytic enzyme, which is highly expressed in a variety of tumor cells, including human breast carcinoma BT-549 cells,human colon cancer cell HCT116, human bladder cancer cell J82 (Tsutsumi et al., 2004, 2009; Funasaka et al., 2009). In lung cancer patients, high PGI expression is associated with poor prognosis and reduced survival rate (Funasaka et al., 2005). The activity of PGI1 can be inhibited by small molecules, i.e., the glucose analog, 2-deoxy-D-glucose (2-DG) (Figure 1) (Pusapati et al., 2016). The small molecule inhibitor, Esculetin can directly bind PGI and repress its activity, which triggers apoptosis and inhibits tumor growth (Wu S. T. et al., 2020).

PGI also acts as an autocrine motility factor (AMF) in tumor progression and metastasis (Funasaka et al., 2005). Its receptor, AMFR/gp78 is overexpressed in multiple tumors and correlated with poor prognosis (Pusapati et al., 2016). It is known that PGI/AMF can function as an anti-apoptotic factor. PGI/AMF down-regulates the expression of apoptotic protease activating factor-1 (Apaf-1) and caspase-9 genes to reduce apoptosis (Haga et al., 2003). In addition, PGI/AMF can reduce apoptosis by activating the PI3K/Akt signaling pathway (Figure 1) (Fruman and Rommel, 2014).

#### Phosphofructokinase 1

Phosphofructokinase 1 (PFK1) catalyzes the conversion of fructose-6-phosphate into fructose-1,6-bisphosphate. PFK1 has 3 isoforms: platelet (PFKP), muscle (PFKM) and liver (PFKL), among which PFKP is the major PFK1 isoform and overexpressed in human glioblastoma cells (Mor et al., 2011). PFK1 facilitates tumor growth as knockdown of PFK1 inhibits the migration and proliferation of colorectal cancer (Lang et al., 2019).

The activity of PFK1 is regulated by different covalent modifications. For example, PFPKP can be acetylated by lysine acetyltransferase 5 (KAT5) at lysine 395 (K395) upon activation of the epidermal growth factor receptor (EGFR) (Lee et al., 2018). Acetylated PFPKP then translocates into the plasma membrane, where it is phosphorylated by EGFR at tyrosine 64 (Y64) to enhance its activity (Lee et al., 2018). PFKP can also been phosphorylated by AKT at serine 386 (S386), which protects PFKP from degradation and facilitates tumor growth (Lee et al., 2017). Under anaerobic conditions, PFK1 is modified by O-linked β-N-acetylglucosamine (O-GlcNAc) at serine 529 (S529) (Yi et al., 2012). Glycosylation of PFK1 reduces its activity and diverts the glucose flux into the pentose phosphate pathway (PPP), resulting in a significant increase in NADPH, which promotes the proliferation and tumorigenicity of lung cancer cells (Yi et al., 2012).

#### Aldolase

Aldolase catalyzes the conversion of fructose 1-6-diphosphate to glyceraldehyde 3-phosphate and dihydroxy-acetone phosphate. Aldolase has three tissue-specific isozymes: ALDOA (muscles), ALDOB (liver) and ALDOC (neuronal tissue) (Lincet and Icard, 2015). Among these isozymes, ALDOA is overexpressed in a series of cancers, including colorectal cancer, hepatocellular carcinoma, lung cancer, and pancreatic cancer and promotes tumor growth, invasion, and migration (Du et al., 2014; Shimizu et al., 2014; Ji et al., 2016; Kawai et al., 2017). Mechanistically, ALDOA activates the EGFR/MAPK pathway to up-regulate the expression of cell cycle protein D1 (cyclin D1) (Fu H. et al., 2018). In lung cancer, ALDOA stabilizes HIF-1α to activate its downstream target MMP9, which enhances lung cancer invasion and migration (Chang et al., 2017). ALDOA also interacts with γ-actin to promote metastasis of non-small cell lung cancer (NSCLC) and blocking this interaction decreases metastasis (Figure 1) (Chang et al., 2019).

Unlike ALDOA, ALDOB is significantly reduced in metastasis-inclined HCC (MIH) and its role in tumorigenesis is ambiguous (Tao et al., 2015). Overexpression of ALDOB inhibits the metastasis of liver cancer cells by inducing Ten-Eleven Translocation 1 (TET1) (Tao et al., 2015). However, another study found that ALDOB is significantly up-regulated by GATA6 in metastatic liver cancer cell HCT116 to accelerate fructose metabolism, which is beneficial to tumor cell proliferation and metastasis (Li et al., 2017). Further efforts are required to elucidate the precise function of ALDOB in tumorigenesis.

#### Glyceraldehyde-3-Phosphate Dehydrogenase

Glyceraldehyde-3-phosphate dehydrogenase (GAPDH) catalyzes the reversible conversion of glyceraldehyde-3-phosphate to 1,3-bisphosphoglycerate. Although GAPDH is a known glycolytic enzyme, it has some non-metabolic functions in tumorigenesis. GAPDH has been reported to increase glycolysis and autophagy to protect cells from caspase-independent cell death (CICD) (Colell et al., 2007). GAPDH accelerates glycolysis to increase ATP production, which is associated with its protective effect from apoptosis in the absence of caspase activation (Colell et al., 2007). Under the same conditions, GAPDH facilitates the expression of Atg12 and increases the autophagic flow, which further preserves cell survival (Colell et al., 2007). Under starvation conditions, GAPDH can inhibit multiple transport pathways including Coat Protein I (COPI) transport by targeting the GTPase-activating protein (GAP) against ADP-Ribosylation Factor 1 (ARF1) (Yang et al., 2018). Consequently, GAPDH reduces energy consumption and maintains energy homeostasis (Yang et al., 2018).

The biological function of GAPDH is closely related to its subcellular location (Tristan et al., 2011). When GAPDH is localized in the cytoplasm, GAPDH can interact with the GTPase Rheb to regulate mTOR and autophagy. During glucose starvation, the interaction between GAPDH and Rheb is enhanced, which dissociates Rheb from mTORC1 to reduce its activity and induce autophagy (Lee et al., 2009). Under high glucose conditions, the glycolytic metabolite glyceraldhyde-3-phosphate interferes with the interaction between GAPDH and Rheb, which promotes mTORC1 activity (Lee et al., 2009). When GAPDH is localized in the mitochondria, GAPDH binds to voltage-dependent anion channels (VDAC) to promote the release of cytochrome *c* and apoptosis-inducing factor (AIF) to induce apoptosis under stress conditions (Tristan et al., 2011). GAPDH has also been reported to translocate into the nucleus when it is S-nitrosated (Kornberg et al., 2010). In the nucleus, GAPDH can interact with the E3 ubiquitin ligase Siah and enhance its activity, leading to Siah-dependent degradation of nuclear proteins and cell death (Hara et al., 2005). The nucleus nitrosylated GAPDH can also interact with the histone deacetylase SIRT1, HDAC2, and DNA-activated protein kinase (DNA-PK) to inhibit their catalytic activity (Kornberg et al., 2010).

GAPDH undergoes various types of covalent modifications, which regulate its localization and/or activity. GAPDH has been reported to be phosphorylated at serine 122 (S122) by AMP-activated protein kinase (AMPK), which promotes its localization in the nucleus (Chang et al., 2015). In the nucleus, the AMPK-phosphorylated GAPDH can competitively bind to SIRT1 and displace SIRT1 repressor DBC1 to activate SIRT1 (Chang et al., 2015). As SIRT1 can stimulate autophagy by deacetylating autophagy protein LC3, the nucleus GAPDH increases autophagy to facilitate cell survival in the absence of glucose (Chang et al., 2015). In addition, GAPDH can be acetylated by histone acetyltransferase p300 and CREB-binding protein CBP, which then enhances the catalytic activity of p300/CBP in a positive feedback manner (Figure 1) (Sen et al., 2008). The nitrosylated GAPDH is translocated to the nucleus and interacts with p300/CBP. Upon acetylation, the nitrosylated GAPDH can deliver the N-Nitroso group to p300/CBP to promote their self-acetylation and enhance their activity (Sen et al., 2008). Activated p300/CBP up-regulates the transcription of their downstream targets, i.e., P53 to inhibit tumor cell proliferation (Tristan et al., 2011).

#### Pyruvate Kinase

Pyruvate kinase (PK) is the last rate limiting enzyme in glycolysis, which catalyzes the production of pyruvate from phosphoenolpyruvate (PEP). PK has four different subtypes L, R, M1, M2 that are encoded by two different genes *PKLR* and *PKM*. The L-type and R-type isozymes are encoded by the gene *PKLR* with differential splicing of RNA. While L-type isoenzyme is mainly expressed in liver, kidney, and intestine, R-type isoenzyme is mainly expressed in erythrocytes (Mazurek, 2011). *PKM* gene can undergo alternative splicing to form PKM1 containing exon 9 or PKM2 containing of exon 10. Although PKM1 can activate glucose catabolism, stimulate autophagy, and promote the proliferation of malignant tumors, including pulmonary neuroendocrine tumors (NETs) and small cell lung cancer (SCLC) (Morita et al., 2018), PKM2 is considered as a specific pyruvate kinase in tumor cells (Israelsen and Vander Heiden, 2015; Morita et al., 2018). PKM2 exists in two states: a dimeric form and a tetrameric form with different PK activities. When PKM2 is in a tetrameric state, it has high PK activity to participate in glycolysis; PKM2 has low PK activity in a dimeric state (Israelsen and Vander Heiden, 2015). Compared with constitutive active PKM1, the activity of PKM2 is low and activated only in the presence of allosteric activators (Chaneton and Gottlieb, 2012). The low PK activity of PKM2 contributes to the “Warburg effect” and metabolic reprogramming in cancer cells. For example, PKM2 diverts glucose metabolism to anabolic metabolism, including the pentose phosphate pathway (PPP), uronic acid pathway (UAP), and polyol pathway (PYP) in a variety of tumor cells and clinical cancer patients, which allows cells to synthesize biological macromolecules (Zhang Z. et al., 2019). The work in yeast revealed that the low PK activity facilitates the diversion of glycolysis to pentose phosphate pathway, which enables cells to accumulate NADPH to maintain the redox balance (Tosato et al., 2012). This implies an important role of PKM2 in tumorigenesis by regulating the antioxidant defense of tumor cells as the strong deregulation of the oxidative stress network has been described in yeast cells with chromosome translocation, which serves as model to study the formation of neoplastic mammalian cells (Sims et al., 2016). In fact, in human lung cancer cells, increased intracellular reactive oxygen species (ROS) concentrations inhibits the glycolytic enzyme pyruvate kinase M2 (PKM2) by oxidating PKM2 at Cys358, which is required to divert glucose flux into the pentose phosphate pathway to combat ROS (Anastasiou et al., 2011).

Due to its important role in tumorigenesis, PKM2 is regulated at both transcriptional and post-transcriptional levels. Three splicing factors, hnRNPL (PTB), hnRNPAI, hnRNPA2 can directly bind exon 9 of *PKM* mRNA, which releases exon 10 and promotes PKM2 expression (Chen et al., 2012). Once activated by epidermal cell growth factor (EGF), the epidermal cell growth factor receptor (EGFR) upregulates the expression of PKM2 by activating the PLCγ1-PKCε-IKKβ-RelA signaling cascade (Yang et al., 2011). PKM2 also undergoes some posttranslational modifications to regulate its activity (Zhang Z. et al., 2019). The acetylation of PKM2 at lysine 305 (K305) reduces its kinase activity by reducing its binding affinity to PEP (Lv et al., 2011). Meanwhile, the acetylation of PKM2 at K305 increases its interaction with heat shock cognate protein 70 (HSC70), which then promotes its degradation by chaperone-mediated autophagy (Lv et al., 2011). The acetylation of KM2 at lysine 433 (K433) inhibits its PK activity by interfering with its binding to allosteric activator FBP (Lv et al., 2013). PKM2 is phosphorylated by fibroblast growth factor receptor type 1 (FGFR1) at tyrosine 105 (Y105), which inhibits its binding to FBP, resulting in a decrease of its activity (Hitosugi et al., 2009).

PKM2 has been reported to function as a transcription cofactor to regulate gene expression. PKM2 can directly interact with the HIF-1α and facilitate the recruitment of HIF-1α and p300 to hypoxia response elements to activate the transcription of HIF-1α target genes (Luo et al., 2011). Nuclear PKM2 binds to β-catenin/TCF/LEF, thereby activating the expression of β-catenin downstream genes, i.e., CCND1 and MYC to promote cell proliferation (Yang et al., 2011). PKM2 forms a complex with pyruvate dehydrogenase (PDC) and p300, which promotes histone acetylation and activates the transcription of aromatic hydrocarbon receptor genes (Figure 1) (Zhang Z. et al., 2019).

Notably, PKM2 has been reported to function as a protein kinase to perform a variety of biological functions. PKM2 phosphorylates the mTORC1 inhibitor AKT1S1 to activate mTORC1 signaling pathway, inhibit autophagy in cancer cells and accelerates oncogenic growth (He et al., 2016). PKM2 inhibits oxidative stress-induced apoptosis by phosphorylating BCL2 to increase its protein stability (Liang et al., 2017). PKM2 phosphorylates the signal transducer and activator of transcription 3 (STAT3) to activate the expression of genes required for tumor cell proliferation (Li et al., 2015) (Figure 1). Nuclear PKM2 phosphorylates β-catenin at tyrosine 333 (Y333) to enhance β-catenin recruitment and dissociate histone deacetylase HDAC3 at the CCND1 promoter region, which eventually promotes CCND1 transcription and cell cycle progress (Yang et al., 2011). PKM2 has also been reported to modulate histone modifications, which will be discussed later.

#### Lactate Dehydrogenase

The LDH family has three isoenzymes: LDHA, LDHB, and LDHC. Among them, LDHA catalyzes the conversion of pyruvate to lactate (Feng et al., 2018); LDHB catalyzes the conversion of lactate to pyruvate (Brisson et al., 2016); LDHC is only expressed in the testis (Hua et al., 2017). LDHA is highly expressed in a variety of tumor cells, includes hepatocellular carcinoma, breast cancer, pancreatic cancer, sophageal squamous cell carcinoma (Sheng et al., 2012; Zhao et al., 2013; Hua et al., 2017; Mohammad et al., 2019; Zhou et al., 2019). Knockout or inhibition of LDHA significantly retards tumor cell proliferation, metastasis, and induces cell apoptosis (Zhuang et al., 2010; Miao et al., 2013), indicating that LDHA plays an important role in tumorigenesis and metastasis.

Under both normal and hypoxic conditions, tumor cells rely heavily on LDHA for energy production (Fantin et al., 2006). LDHA may increase the carcinogenicity of intestinal-type gastric cancer (ITGC) by indirectly regulating the expression of octamer-binding transcription factor 4 (Oct-4) (Zhang et al., 2012). LDHA also protects tumor cells from ROS and apoptosis (Le et al., 2010).

The activity of LDHA can be regulated by post-translational modifications. LDHA is phosphorylated at tyrosine 10 (Y10) by oncogenic receptor tyrosine kinase FGFR1, which facilitates the formation of active LDHA tetramers (Fan et al., 2011). FGFR1-catalyzed LDHA phosphorylation can be enhanced by adenylate kinase hCINAP to augment the catalytic activity of LDHA (Ji et al., 2017). LDHA can also be acetylated and recognized by HSC70, which then delivers LDHA to lysosome to be degraded (Zhao et al., 2013).

LDHB is also essential for tumorigenesis and tumor cell survival, which is associated with its regulatory role in autophagy. Protons generated by LDHB is necessary to promote lysosomal acidification and maintain basal autophagy in cancer cells (Brisson et al., 2016). LDHB can be phosphorylated by Aurora-A to promote glycolysis and tumor progression (Cheng et al., 2019). In addition, LDHB is acetylated at lysine 329 (K329), which can be deacetylated by SIRT5 to enhance its activity (Shi et al., 2019). SIRT5-mediated LDHB deacetylation promotes autophagy and tumorigenesis (Shi et al., 2019).

### Gluconeogenesis Enzymes and Tumorigenesis

The glucose concentration in the central area of solid tumors is low (Butler et al., 1975). In the absence of glucose, tumor cells need to use smaller carbon substrates such as lactate and amino acids to synthesize glucose and precursors required for tumor cell growth by gluconeogenesis (Hu et al., 2017). Therefore, the key enzymes in the process of gluconeogenesis are also critical to cancer occurrence and development.

#### Pyruvate Carboxylase

Pyruvate carboxylase (PC) not only provides oxaloacetate for gluconeogenesis, but also participates in the *de novo* synthesis of fatty acids and the synthesis of glycerol (Jitrapakdee et al., 2006). PC catalyzes the conversion of pyruvate to oxaloacetate, which is then condensed with acetyl-CoA to form citrate. The PC-mediated pyruvate/citrate cycle is necessary for *de novo* fatty acid synthesis (Ballard and Hanson, 1967; Kajimoto et al., 2005). Glycerol is also derived from oxaloacetate with the coordinated action of PC and phosphoenolpyruvate carboxykinase (PEPCK). PEPCK converts oxaloacetate to phosphoenolpyruvate (PEP), which is subsequently converted to glycerol (Reshef et al., 2003).

PC is overexpressed in human breast cancer tissues and correlated with the late stage of tumor progression (Phannasil et al., 2015). PC is important to support the growth and invasion of breast cancer (Phannasil et al., 2015). In addition to anaplerosis, PC affects pyruvate cycling and biosynthesis of metabolites such as the serine, glycine, 5-carbon sugar, and fatty acids (Phannasil et al., 2017).

#### Phosphoenolpyruvate Carboxykinase

Phosphoenolpyruvate carboxykinase (PEPCK) decarboxylates and phosphorylates oxaloacetate to form PEP in the second step of gluconeogenesis. PEPCK has two isozymes: PEPCK1 (encoded by *PCK1*), PEPCK2 (encoded by *PCK2*), which are distributed in the mitochondria and cytoplasm, respectively (Figure 1). PEPCK1 is overexpressed in colorectal cancer and melanoma (Li Y. et al., 2015), whereas PEPCK2 is highly expressed in lung cancer (Vincent et al., 2015), prostate cancer (Zhao et al., 2017), and breast cancer (Mendez-Lucas et al., 2014).

The effect of PEPCK on tumorigenesis is tissue-specific. Overexpression of PEPCK1 activates mTORC1 to promote glycolysis and facilitate colorectal cancer proliferation (Vincent et al., 2015). Under the conditions of glucose deficiency, amino acid limitation, and endoplasmic reticulum (ER) stress, the transcription of PEPCK2 is up-regulated, thereby increasing the adaptability of breast cancer cells to stress conditions (Morita et al., 2018). However, PEPCK1 has been reported to be down-regulated and function as a tumor suppressor in clear cell renal cell carcinoma (ccRCC), liver cancer and hepatocellular carcinoma (HCC) (Tuo et al., 2018, 2019; Shi et al., 2020). Overexpression of PEPCK1 in liver cancer cells significantly reduces the level of cellular ATP to activate AMPK and promote the expression of p27, which causes cell cycle arrest at G1 phase (Tuo et al., 2019). Knockdown of PEPCK1 in ccRCC cells increases the stability of LDHA to enhance the “Warburg effect” and promote cell proliferation and metastasis (Shi et al., 2020). Overexpression of PCK1 can inhibit proliferation of hepatoma cells by reducing the production of ROS and repressing the expression of thioredoxin reductase 1 (TXNRD1) (Tuo et al., 2018).

#### Fructose-1,6-Bisphosphatase

Fructose-1,6-bisphosphatase (FBPase) catalyzes the second rate-limiting reaction of gluconeogenesis. There are two isoforms of FBPase in mammals: FBP1 (liver) and FBP2 (muscle). Both FBP1 and FBP2 function as tumor suppressors to inhibit the proliferation of tumor cells. Depletion of FBP1/2 contributes to the initiation, promotion and progression of a variety of tumors, including pancreatic ductal adenocarcinoma (PDAC) (Moore et al., 2012), ccRCC (Hirata et al., 2016), colon cancer (Zhang et al., 2016), gastric cancer (Bigl et al., 2008), lung cancer (Gutteridge et al., 2017), and cervical carcinoma (Zhang Y. P. et al., 2019). The FBP1 promoter region in tumor cells is hypermethylated to silent its expression, which is beneficial to tumor cell survival and proliferation (Gutteridge et al., 2017). Knockdown of FBP1 can promote the epithelial-mesenchymal transition, invasion, and metastasis of prostate cancer cells by activating the MAPK signaling pathway, while overexpression of FBP1 can inhibit the proliferation and metastasis of cholangiocarcinoma cells through the Wnt/β-catenin pathway (Figure 1) (Zhao et al., 2018). FBP2 has also been reported to activate the AMPK signal transduction, inhibit the Akt-mTOR pathway, attenuate glucose metabolism, enhance cell apoptosis, and inhibit tumor cell proliferation (Figure 1) (Li et al., 2013).

#### Glucose-6-Phosphatase

Glucose-6-phosphatase (G6Pase) catalyzes the last step of gluconeogenesis, which consists of glucose-6-phosphatase catalytic subunit (G6PC) and glucose-6-phosphate translocase (G6PT). G6PT transports glucose-6-phosphate (G-6-P) from the cytoplasm into the endoplasmic reticulum (ER) lumen, where G-6-P is hydrolyzed by G6PC. G6Pase is overexpressed in non-gluconeogenic tumors such as ovarian cancer and glioblastoma (Wang and Dong, 2019). In G6PC-rich ovarian cancer and glioblastoma, G6PC facilitates tumor growth by promoting glycogenolysis. Knockdown of G6PC activates the expression of glycogen synthase and reduces the expression of glycogen phosphorylase, leading to accumulation of glycogen and cell cycle arrest, thereby inhibiting tumor cell proliferation and triggering cell death (Wang and Dong, 2019). In addition, G6PC positively regulates cell cycle progression by modulating cyclin dependent kinase inhibitor 1B (CDKN1B) (Guo et al., 2015). Inhibition of G6PC can enhance the phosphorylation of CDKN1B, thereby reducing the expression of CDK2 and cyclin D1, leading to cell cycle arrest (Guo et al., 2015). G6PT can also promote the invasion of glioblastoma in combined with membrane type 1 matrix metalloproteinase (MT1-MMP) (Fortier et al., 2008). Inhibition of G6PT and MT1-MMP blocks the MAPK pathway and prevents brain tumor invasion (Figure 1) (Fortier et al., 2008). Under hypoxia conditions, knockout of G6PT induces necrosis of glioblastoma cells (Lord-Dufour et al., 2009). However, in glycemic-rich tissues such as liver, G6PC mainly acts on gluconeogenesis and glycogen accumulation, which is not beneficial for tumorigenesis. Thus, G6PC is often expressed at low levels in liver cancer tissues to accumulate a large amount of G-6-P, which could be used for nucleotide synthesis (Wang et al., 2012).

### TCA Cycle and Tumorigenesis

TCA cycle is the central hub of energy metabolism, macromolecule synthesis, and redox balance. Enzymes in the TCA cycles play important roles in cancer metabolism and tumorigenesis (Anderson et al., 2018). In this section, we will describe the following TCA enzymes: citrate synthase (CS), isocitrate dehydrogenase (IDH), α-ketoglutarate dehydrogenase complex (α-KGDHC), and succinate dehydrogenase (SDH).

#### Citrate Synthase

**Figure 2:**
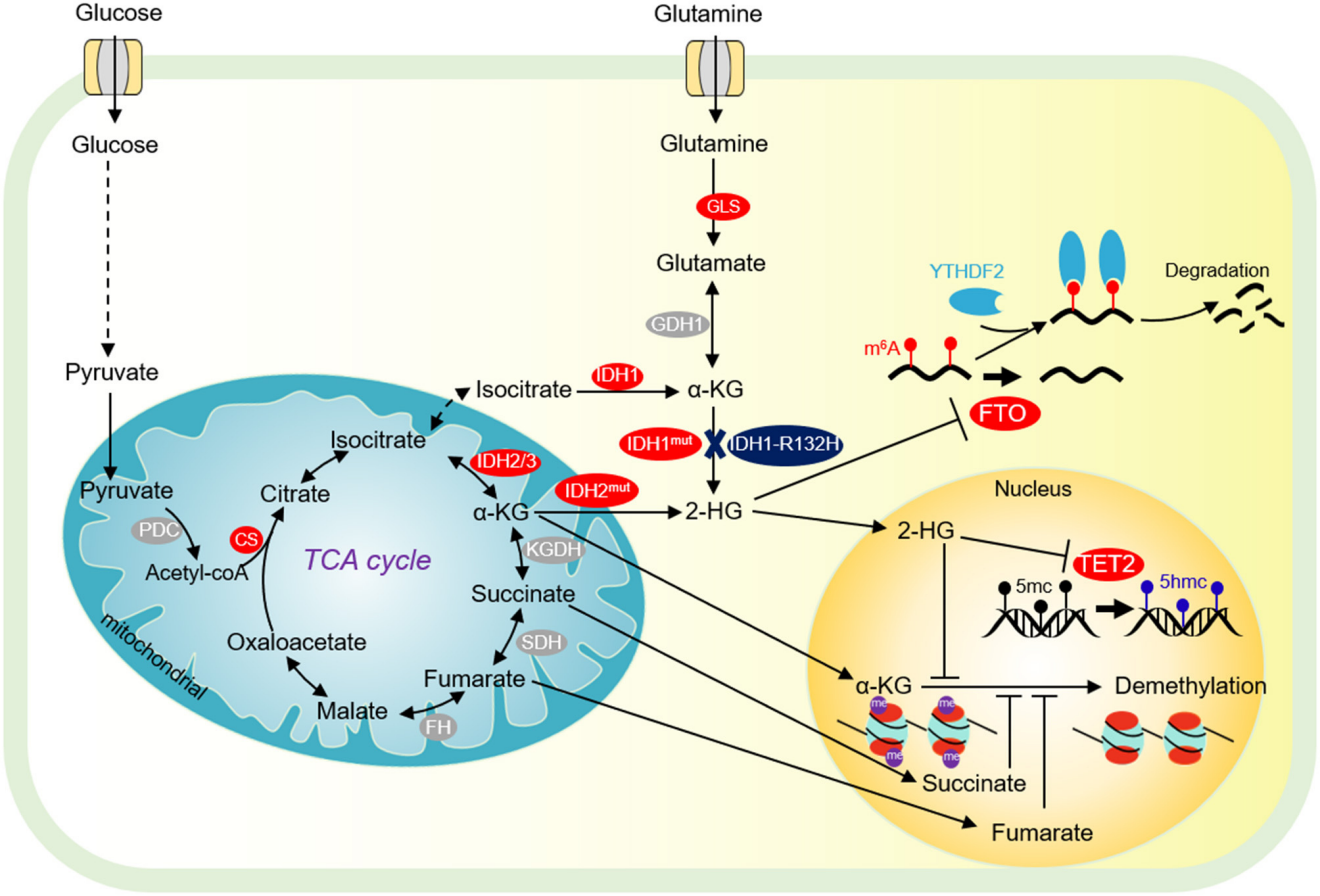
Tricarboxylic acid (TCA) cycle and chromatin demethylation in cancer cells. Although the TCA cycle is repressed in tumor cells, it is also essential for tumor growth. α-KG derived from glucose metabolism or glutamine is essential for tumor cell proliferation and chromatin demethylation. α-KG can be metabolized to succinate and fumarate by FH and SDH in the TCA cycle, respectively. Succinate and fumarate are competitive inhibitors of α-KG dependent demethylases. α-KG can also be converted to 2-HG by mutated isocitrate dehydrogenase 1/2 (IDH1/2). 2-HG is a competitive inhibitor of α-KG-dependent demethylase, which can inhibit DNA methylation mediated by TET2 and JMJD2D, and FTO-mediated m^6^A-dependent mRNA degradation. PDC, pyruvate dehydrogenase complex; CS, citrate synthase; FH, fumarate dehydrogenase; SDH, succinate dehydrogenase; IDH1/2/3, isocitrate dehydrogenase 1 and 2 and 3; Mut IDH1/2, mutated isocitrate dehydrogenase 1 and 2; α-KG, α-ketoglutarate; FTO, fat mass- and obesity-associated; YTHDF2, YTH domain-containing family protein 2.

Citrate synthase (CS) is the first rate-limiting enzyme in the TCA cycle, catalyzing the formation of citrate from acetyl-CoA and oxaloacetate (Figure 2). Growing evidence indicates that CS plays an important role in regulating cancer cell growth and tumorigenesis (Chen L. et al., 2014). Knockdown of CS inhibits tumor cell proliferation, cell colony formation, cell migration, cell invasion, and cell cycle progression (Cai et al., 2020).

The expression of CS is regulated by STAT3. Inhibition of STAT3 down-regulates the expression of CS and thus inhibits cell growth and proliferation (MacPherson et al., 2017). Exogenous addition of sodium citrate attenuates this inhibitory effect (MacPherson et al., 2017).

#### Isocitrate Dehydrogenase

Isocitrate dehydrogenase (IDH) is the second rate-limiting enzyme in the TCA cycle. IDH is composed of three isoenzymes: the cytoplasmic IDH1 and the mitochondrial IDH2/3 (Figure 2). IDH is mutated in a variety of tumor cells (Sajnani et al., 2017). IDH mutations stimulate the occurrence of cancer in a variety of biological processes. Tumor cells with IDH1R132H mutations can increase the level of HIF-1α, thereby promoting tumor cell growth (Figure 2) (Zhao et al., 2009). The IDH1 R132H mutant catalyzes the conversion of α-KG to oncometabolite 2-hydroxyglutamate (2-HG) (Figure 2). The mutations of R172 and R140 in IDH2 lead to accumulation of 2-HG at extremely high levels (Dang et al., 2016). High levels of 2-HG competitively inhibit the activities of α-KG-dependent histone demethylase and TET2 DNA demethylase, leading to global chromatin methylation disorders, which is beneficial to tumorigenesis (Chowdhury et al., 2011; Xu et al., 2011). Interestingly, 2-HG has also been reported to repress tumor cell proliferation by targeting FTO/m^6^A/MYC/CEBPA signals (Figure 2) (Su et al., 2018). R-2HG targets Fat mass and obesity-associated (FTO) and inhibits its RNA demethylase activity, which significantly increases the abundance of m^6^A in cancer cells, leading to degradation of myc/CEBPA mRNAs in an m^6^A-dependent manner (Su et al., 2018). As a consequence, tumor cell proliferation is impaired (Su et al., 2018). Inhibition of FTO by R-2-HG also ameliorates aerobic glycolysis by increasing m^6^A levels in the mRNA of phosphofructokinase (PFKP) and LDHB, which reduces the expression of PFKP and LDHB (Qing et al., 2021).

#### α- Etoglutarate Dehydrogenase Complex

α-ketoglutarate dehydrogenase complex (α-KGDHC) is the third rate-limiting enzyme in the TCA cycle, which is composed of α-KG dehydrogenase (OGDH), dihydrolipidamide S-succinyltransferase (DLST) and dihydrolipidamide dehydrogenase (DLD). The expression of OGDH is low in many cancers due to DNA hypermethylation at its promoter and the low expression of DLD is related to the poor prognosis of some cancer patients (Zhang et al., 2015; Shin et al., 2020). Recent studies have shown that the α-KGDH complex enters into the nucleus and acts with lysine acetyltransferase 2A (KAT2A) to catalyze histone succinylation, which is beneficial for cell proliferation and tumor development (Figure 2) (Wang et al., 2017).

#### Succinate Dehydrogenase

Succinate dehydrogenase (SDH) converts succinate to fumarate. Mutations or loss of function of SDH can lead to hereditary paraganglioma, pheochromocytoma, and other genetic syndrome (Sajnani et al., 2017). The single-base substitution of SDH that causes a 33-amino acid C terminal truncation of SDHC protein, increases the intracellular ROS, which leads to metabolic stress, genome instability, and promote tumorigenesis (Slane et al., 2006). Meanwhile, SDH mutation can lead to the accumulation of succinate, which can activate succinate receptor 1 (SUCNR1), also known as regulatory G protein coupled metabolic receptor 91 (GPR91), and stimulate the release of vascular endothelial growth factor (VEGF), which then activates various kinase signal transduction pathways involved in tumorigenesis, angiogenesis, and other biological processes (Sajnani et al., 2017).

## Impact of Glucose Metabolites on Tumorigenesis

**Figure 3:**
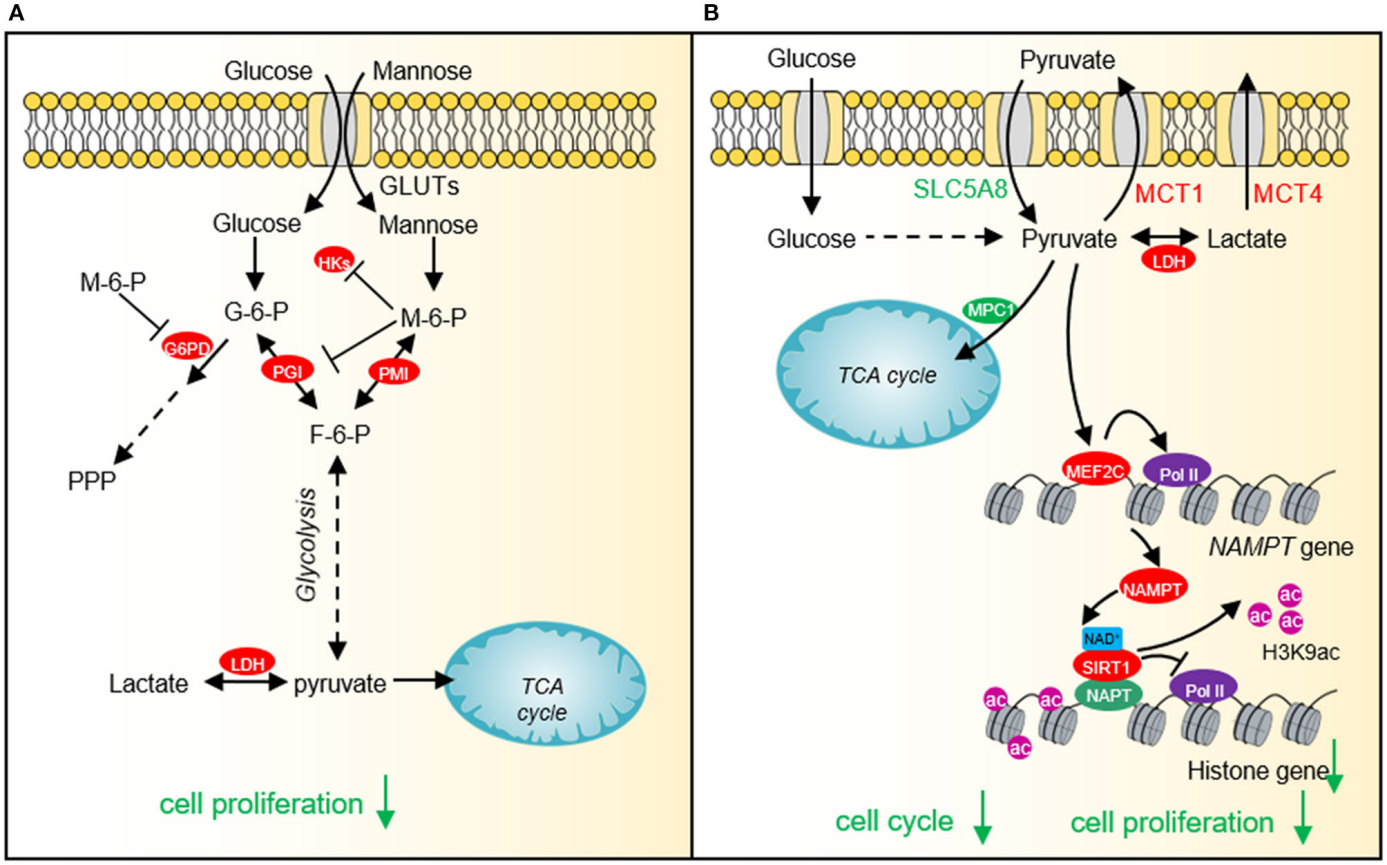
The effect of mannose and pyruvate on cancer cell proliferation. **(A)** Exogenous mannose generates mannose-6-phosphate under the catalysis of hexokinase. High levels of mannose-6-phosphate significantly inhibit aerobic glycolysis and pentose phosphate pathway (PPP) by inhibiting the activities of hexokinase, PGI and G6PD, which affects lactate production and TCA cycle, resulting in cell growth arrest and chemotherapeutic sensitivity. **(B)** Pyruvate represses histone gene expression by inducing the expression of NAD^+^ biosynthesis enzyme, nicotinamide phosphoribosyltransferase (NAMPT) *via* myocyte enhancer factor 2C (MEF2C), which then increases NAD^+^ levels and activates the histone deacetylase activity of SIRT1. Downregulated histone gene expression leads to cell cycle arrest and tumor cell growth defects. HKs, hexokinases; G-6-P, glucose-6-phosphate; M-6-P, Mannose-6-phosphate; PMI, Mannose-6-phosphate isomerase; PGI, phosphoglucose isomerase; F-6-P, fructose-6-phosphate; LDH, lactate dehydrogenase; SLC5A8, Sodium-coupled monocarboxylate transporter 1; MCT1, Monocarboxylate transporter 1; MCT4, Monocarboxylate transporter 4; MEF2C, myocyte enhancer factor 2C; NAMPT, nicotinamide phosphoribosyltransferase; SIRT1, Silent mating type information regulation 2 homolog-1; NPAT, Nuclear protein of the ataxia telangiectasia mutated locus.

In order to fulfill the needs of tumor cell growth and proliferation, tumor cells must increase the uptake of nutrients. Glucose and glutamine are the two main nutrients used for cell proliferation and biomacromolecule synthesis in tumor cells (Pavlova and Thompson, 2016). Lactate has recently been shown to serve as an energy source to help tumor cells survive in the absence of glucose (Rabinowitz and Enerback, 2020). Therefore, glucose-derived intermediate metabolites have become the targets of cancer treatment. Limiting the glucose uptake or repressing the expression and activity of glucose transporter GLUT1 can significantly inhibit the proliferation and survival of a variety of tumor cells (Gras et al., 2014). Mannose, as a monosaccharide, shares the same transporter with glucose, which significantly inhibits the growth of various tumor cells and the proliferation of xenogeneic tumors in nude mice (Figure 3A) (Gonzalez et al., 2018). 2-DG is a glucose analog, which is also transported by GLUT into cells and then phosphorylated by hexokinase to form 2-deoxy-D-glucose-6-phosphate (2DG-6-P) (Zhang et al., 2014). The accumulation of 2DG-6-P attenuates glycolysis by non-competitively inhibiting HK and competitively inhibiting PGI, which in turn leads to reduced ATP production, cell cycle arrest, and cell death (Figure 1) (Zhang et al., 2014).

### Pyruvate

Our recent study shows that cancer cells prefer low intracellular pyruvate as the intracellular pyruvate concentration is lower in cervical cancer and lung cancer than adjacent normal tissues (Ma et al., 2019). Exogenous supplied pyruvate inhibits the proliferation of various types of cancer cells, including human cervical cancer cells, liver carcinoma cells, and breast adenocarcinoma cells pyruvate as well as xenografted tumor, implying that pyruvate can function as a potential anti-cancer compound (Ma et al., 2019). Further study indicates that pyruvate reduces the expression of core histone genes to decompact chromatin structure and cause cell cycle arrest (Figure 3B) (Ma et al., 2019). Mechanistically, pyruvate induces the expression of NAD^+^ biosynthetic enzyme nicotinamide phosphoribosyl transferase (NAMPT) by activating myocyte enhancer factor 2C (MEF2C). The up-regulated NAMPT significantly promotes the biosynthesis of NAD^+^, thereby enhancing the activity of the NAD^+^-dependent histone deacetylase SIRT1. The activated SIRT1 reduces H3K9ac in the promoter region of histone genes and represses histone gene transcription (Ma et al., 2019).

The pyruvate analogs have also been reported to have anti-cancer effects. 3-bromopyruvate not only effectively attenuates aerobic glycolysis of tumor cells, but also inhibits mitochondrial oxidative phosphorylation (Fan et al., 2019; Linke et al., 2020). Ethyl pyruvate, another derivative of pyruvate, has recently been reported to inhibit tumor cell invasion, migration, and apoptosis (Liu Q. et al., 2019; Zhang T. et al., 2019). Specifically, ethyl pyruvate inhibits the growth of non-small cell lung cancer (NSCLC) by blocking the high mobility group box 1 (HMGB1)-RAGE axis and NF-κB/STAT3 pathway (Liu Q. et al., 2019). Ethyl pyruvate down-regulates the expression of HMGB1 and RAGE, induces the accumulation of cell cycle inhibitor p27, which leads to blockage of cell cycle progression (Liu Q. et al., 2019; Zhang T. et al., 2019).

### Lactate

Increased lactate production has been considered as an important marker of the “Warburg effect.” Lactate not only acts as an energy source and gluconeogenic precursor to protect cancer cells from glucose deprivation, but also functions as a signaling molecule to mediate a variety of biological processes (Hu et al., 2017; Brooks, 2020). As a shuttle molecule, lactate plays an important role in converting oxidative cells to glycolytic cells. Lactate inhibits prolylhydroxylase 2 activity and activates HIF-1α in normoxic oxidative tumor cells. HIF-1α then up-regulates the transcription of glycolytic enzymes, which accelerates glycolysis and promotes tumor growth (De Saedeleer et al., 2012). In addition to HIF-1α, lactate also affects the transcription of key oncogenes (MYC, RAS, PI3KCA), transcription factors (E2F1), tumor suppressors (BRCA1, BRCA2) and genes involved in cell cycle and proliferation (San-Millan et al., 2019). Lactate can also stabilize HIF-2α and activate MYC to induce glutamine transporter ASCT2 and glutaminase 1 (GLS1). The high expression of GLS1 promotes the uptake and catabolism of glutamine, which is beneficial for the survival and proliferation of oxidative cancer cells (Brooks, 2020). Another important mechanism by which lactate regulates gene expression is causing lactylation of lysine residues on histones, which will be discussed later. Therefore, targeting lactate metabolism or inhibiting lactate uptake has become an effective strategy for tumor treatment (Brooks, 2020).

### Citrate

Citrate has potential anti-tumor functions in different types of cancer cells, including acute monocytic leukemia (U937), breast cancer (MCF-7), pancreatic cancer (BxPC3), lung cancer (A549), Glioma (G6), neuroblastoma (Tet21N), pleural mesothelioma (MSTO-211H), gastric cancer BGC-823 and SGC-790 (Huang et al., 2020). Sodium citrate can attenuate glycolysis and cause cell cycle arrest by inhibiting the activity of PFK1 (Huang et al., 2020). Citrate can also reduce the expression of anti-apoptotic proteins B-cell lymphoma-2 (Bcl-2) and Myeloid cell leukemia-1 (Mcl-1) to trigger cell apoptosis (Chen L. et al., 2014).

## Metabolic Regulation of Chromatin Modifications

Most chromatin modifying enzymes use metabolites as cofactors or substrates, so their activity is directly or indirectly regulated by these metabolites, such as acetyl coenzyme A (acetyl-CoA), S-adenosylmethionine (SAM). In addition, α-ketoglutarate, lactate, succinate, and some short chain fatty acids have recently been found to be involved in chromatin modifications. In this section, we will discuss the effects of glucose-derived metabolites and metabolic enzymes on chromatin modifications.

### Contribution of Metabolism to Chromatin Modification in Cancer

#### Histone Glcnacylation

Acetylglucosamine glycosylation (GlcNAcylation) is one of the most common protein post-translational modifications. It is an advanced glycation formed by a series of condensation, oxidation, and rearrangement of monosaccharides in the form of aldose (such as glucose and fructose) or glycolysis by-products (Dall'olio and Trinchera, 2017). GlcNAcylation can directly affect chromatin structure and gene expression by modifying histone residues or epigenetic regulatory factors (histone methyltransferases, protein kinases, and acetyltransferases) (Slawson et al., 2008; Sakabe and Hart, 2010; Dehennaut et al., 2014). Glycosylated histones H3 and H4 disrupt the assembly of nucleosomes and reduce histone acetylation, leading to disassembly of chromatin structure and deregulated gene expression (Chen et al., 2013). Breast cancer cells have a high level of histone H3 glycosylation; however, when the deglycase DJ-1 is knocked down, the overall level of histone glycosylation is significantly increased and the cell viability is decreased (Zheng et al., 2019). This result indicates that when histones have excessive glycosylation, it greatly affects histone acetylation and ubiquitination and causes damage to tumor cells. DNA demethylase TET2 interacts with glycosyltransferase OGT and forms a complex at the transcription start site (TSS) to induce histone H2B S112 GlcNAcylation and promote gene transcription (Chen et al., 2013). In addition, multiple amino acid residues of histone demethylase EZH2 can be glycosylated by OGT to enhance its protein stability and enzymatic activity to promote tumor progression (Lo et al., 2018).

#### Histone Phosphorylation

**Figure 4:**
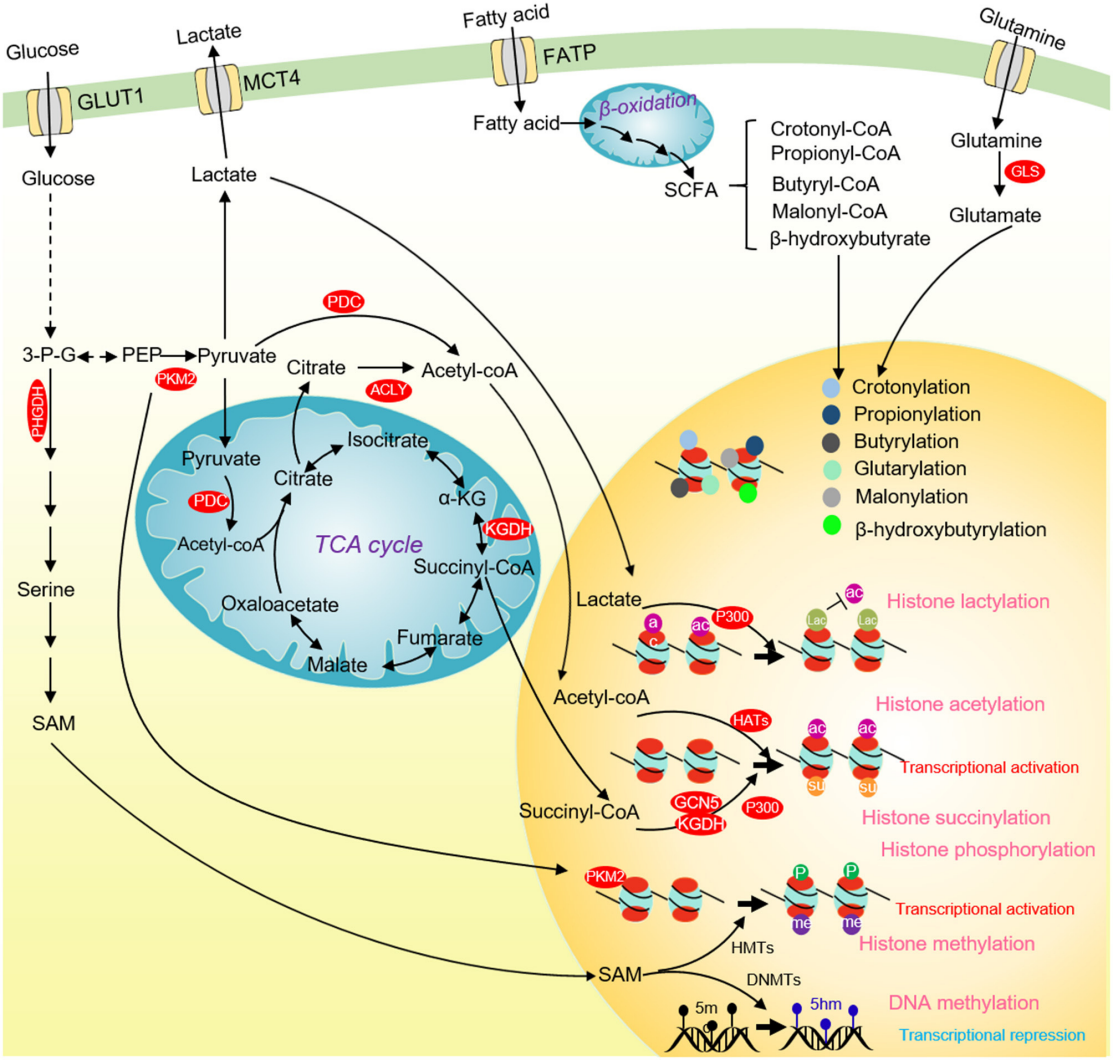
Glucose metabolism and chromatin modifications in cancer cells. ACLY and PDC contribute to nuclear acetyl-CoA production and subsequent histone acetylation and gene activation. Glucose-derived serine and one-carbon metabolism are accelerated in cancer cells to produce SAM. SAM not only enhances DNA methylation to repress the transcription of tumor suppressor genes, but also activates the transcription of tumor-promoting genes by methylating histones. Nuclear PKM2 phosphorylates STAT3 to activate CCND1 transcription and promote cell cycle progression. The TCA cycle intermediate succinate-CoA is fueled to histone succinylation by succinyltransferase (α-KGDH, GCN5, and p300) to activate gene transcription. p300 uses lactate as a substrate to catalyze the lactylation of multiple histone lysine residues to inhibit histone acetylation. Some short-chain fatty acids produced by β-oxidation of fatty acids can provide substrates for histone crotonylation, propionylation, butyrylation, β-hydroxyisobutyrylation, glutarylation and malonylation. ACLY, ATP-citrate lyase; PKM2, pyruvate kinase M2; α-KG, α-ketoglutarate; SCFAs, short-chain fatty acids; 3-P-G, 3-phosphoglycerate; SAM, S-adenosyl methionine; HMTs, Histone methyltransferases; DNMTs, DNA methyltransferases; GCN5, general control non-derepressible 5; p300, histone acetyltransferase p300.

The direct evidence to link glucose metabolism and histone phosphorylation is the finding that PKM2 phosphorylates H3 on threonine 11 (H3T11) (Yang et al., 2012). PKM2 interacts with β-catenin and binds at the CCND1 promoter region to phosphorylate histone H3T11, which causes the dissociation of histone deacetylase HDAC3 from chromatin and an increase of H3K9 acetylation (H3K9ac) at CCND1 promoter to activate CCND1 transcription (Figure 4) (Yang et al., 2012). Our group also showed that the yeast pyruvate kinase Pyk1 can form a complex called SESAME to phosphorylate histone H3T11 (H3pT11) to repress gene expression in response to glucose and serine availability (Li S. et al., 2015; Yu et al., 2017). We also find that SESAME phosphorylates H3T11 at telomeres to maintain the integrity of telomere heterochromatin during chronological aging (Zhang et al., 2021). H3pT11 can contribute to tumorigenesis by recruiting chromatin modifying factors. For example, H3pT11 can be bound by WDR5, a subunit of MLL complex to promote H3K4 methylation (Kim et al., 2014). In addition, the E3 ubiquitin ligase RNF8 can bind H3pT11 to mediate H3K4 ubiquitination and facilitate the expression of MYC and CCND1 (Xia et al., 2017).

#### Histone Acetylation

Acetyl-coenzyme A (acetyl-CoA) is an important substrate for histone acetylation. Acetyl-CoA is produced from pyruvate, citrate, and acetate by pyruvate dehydrogenase complex (PDC), ATP citrate synthase (ACLY), and acetyl-CoA synthetase short-chain family member (ACSS), respectively. Acetyl-CoA can also be generated from fatty acid β-oxidation as well as the metabolism of amino acids and ketone bodies (Sivanand et al., 2018). In cancer cells, deprivation of glucose, glutamine or pyruvate reduces the overall level of histone acetylation (Mcbrian et al., 2013). The glycolysis inhibitor 2-DG or knockdown of hexokinase, pyruvate dehydrogenase, and pyruvate kinase significantly reduces most histone acetylation, leading to compacted chromatin structure, repressed gene expression and reduced tumor cell proliferation (Liu et al., 2015; Yucel et al., 2019). These studies indicate that glucose metabolism can directly regulate histone acetylation.

In addition to modulating the intracellular acetyl-CoA level, glucose metabolism can affect histone acetylation by regulating the activity of histone deacetylase enzyme HDAC. The glucose-derived pyruvate can inhibit tumor cell proliferation by activating the HDAC activity of SIRT1 to reduce H3K9ac (Ma et al., 2019). The catalytic activity of class III histone deacetylases (Sirtuins) depends on NAD^+^, a cofactor of glucose metabolism, which makes it sensitizes to NAD^+^ and glucose metabolism changes. Moreover, the activity of Sirtuins is regulated by some NAD^+^ producing enzymes. The nuclear LDH interacts with SIRT1 and enhances its activity by producing localized NAD^+^, which helps cells adapt to oxidative stress (Castonguay et al., 2014). GAPDH interacts with Sir2, the yeast homolog of SIRT1, and enhances its activity by elevating nuclear NAD^+^ (Ringel et al., 2013).

#### Histone Lactylation

Recent studies revealed that histones can undergo a new type of histone modification, histone lactylation (Figure 4) (Zhang et al., 2019). As a product of glucose metabolism, lactate serves as the substrate for histone lactylation, which can modify at ~28 residues on human and mouse core histones (Zhang et al., 2019). Like histone acetylation, the lactylated histones can directly promote gene transcription (Zhang et al., 2019). The discovery of histone lactylation provides a new insight into studying the role of lactate in tumorigenesis and metabolic diseases.

#### Chromatin Methylation

Chromatin methylation consists of three major types: DNA, RNA, and histone methylation. Chromatin methylation is catalyzed by methyltransferases using SAM as a methyl donor, which is mainly produced from glucose-derived serine metabolism (Yu et al., 2018). We and others have reported that the intracellular SAM concentration can specifically regulate the level of histone H3K4 trimethylation (H3K4me3) in both yeast and mouse embryonic stem cells (mESCs) (Figure 4) (Shyh-Chang et al., 2013; Wu et al., 2019). As a methyl donor, the intracellular SAM also regulates the global DNA methylation. The *de novo* DNA methylation by DNA methyltransferase 3a (DNMT3a) is sensitive to SAM changes, and SAM can effectively induce DNA methylation and reduce the expression of Vascular endothelial growth factor C (VEGF-C), thus inhibiting tumor growth (Nishikawa et al., 2015).

#### Chromatin Demethylation

It has been reported that α-ketoglutarate (α-KG) acts as a cofactor for several chromatin-modifying enzymes, including histone demethylase, Ten-eleven-translocation (TET) family of enzymes involved in DNA and RNA demethylation (Figure 2) (Tran et al., 2019). α-KG is an important metabolic intermediate produced in the TCA cycle, and is a key point linking carbon and nitrogen metabolism. Recent studies show that inhibition of glucose metabolism reduces α-KG level and inhibits the activity of the Jumonji family demethylase JMJD2B (Fu L. N. et al., 2018). JMJD2B can demethylate H3K9/K36 at autophagy associated gene (*ATG*) promoter to regulate autophagy (Fu L. N. et al., 2018). The glucose-derived α-KG maintains H3K27me3 at low levels to regulate chromatin accessibility and glioma cell survival (Chung et al., 2020). The TET proteins are α-KG-dependent dioxygenases that oxidize 5-methylcytosine (5mC) to 5-hydroxymethylcytosine (5hmC) to mediate DNA demethylation (Xiao et al., 2012). α-KG can regulate genome-wide DNA methylation and tumorigenesis by targeting TET (Xiao et al., 2012). In addition, the RNA demethylase, Fat mass and obesity-associated protein (FTO) is also α-KG-dependent (Jia et al., 2011). Therefore, emerging researches for cancer therapy are aimed at manipulating the α-KG level.

As described earlier, α-KG can be converted to 2-HG by the IDH1/2 mutant. 2-HG is also a competitive inhibitor of multiple dioxygenases (Wang P. et al., 2015). R-2HG can inhibit the RNA demethylase activity of FTO, increase m^6^A at *MYC* and *CEBPA* mRNAs and reduce their stability, which ultimately inhibits tumor growth (Su et al., 2018). Thus, α-KG plays an important role in regulating chromatin (de)methylation by targeting the activity of chromatin demethylases. In addition, glycolysis can also indirectly inhibit the activity of histone demethylase Jhd2 by promoting H3K14ac, thus mediating a crosstalk between H3K14ac and H3K4me3 (Wu et al., 2019).

#### Histone Acylation

Histone acylation is an important chromatin modification for transcription regulation (Figure 4). There are various types of histone acylation, including crotonylation, 2-hydroxyisobutyrylation, butyrylation, propionylation, glutarylation, malonylation, and succinylation (Yu et al., 2018). These modifications are regulated by short-chain acyl-CoA species, which are produced during cellular metabolism. Histone acylation is associated with transcriptionally active genes and functions in a variety of physiological processes, including signal-dependent gene activation, spermatogenesis, tissue injury, and metabolic stress (Sabari et al., 2017). The histone acetyltransferase, p300 has crotonyltransferase activity and catalyzes H3K18 crotonylation depending on the intracellular crotonyl-CoA concentrations (Kollenstart et al., 2019). Chromodomain Y-like corepressor CDYL acts as a crotonyl-CoA hydratase to convert crotonyl-CoA to β-hydroxybutyryl, which negatively regulates histone crotonylation during spermatogenesis (Liu et al., 2017). Under chronic social defeat stress, CDYL is upregulated to reduce histone crotonylation and increase H3K27 trimethylation (H3K27me3), leading to repressed transcription of neuropeptide VGF (Liu Y. et al., 2019). The class I histone deacetylases (HDACs) have been demonstrated to function as the major histone decrotonylases and are regulated by microbiota derived short chain fatty acids (Wei et al., 2017).

Histone propionylation is an active chromatin marker. Propionyl-CoA stimulates gene transcription by promoting the enrichment of H3K14 propionylation at promoters of active genes (Kebede et al., 2017). The MYST family of lysine acetyltransferases (KATs) MOF directly binds propionyl-CoA and possesses strong propionyltransferase activity both *in vitro* and *in vivo* (Han et al., 2018). Histone acetyltransferase p300 can also catalyze H3K23 propionylation and histone deacetylase SIRT2 can remove this modification in presence of NAD^+^, which mediates the crosstalk between histone acetylation and propionylation (Liu et al., 2009).

Similarly, histone butyrylation is catalyzed by p300/CBP and regulated by intracellular butyryl-CoA level (Chen et al., 2007). Histone K5 and K8 butyrylation is enriched at meiotic gene promoter and mediates spermatogenesis (Du et al., 2011).

Histone glutarylation and malonylation are recently identified histone modifications despite little is known about their biological functions. H4K91 can be glutarylated by lysine acetyltransferase 2A (KAT2A) and deglutarylated by SIRT7, which has been served as a new regulatory mechanism for chromatin dynamics (Bao et al., 2019). Molonylation of histone H2AK119 can inhibit H2A phosphorylation and influence chromosome segregation (Ishiguro et al., 2018). Histone malonylation also provides a new insight into neural tube defects (NTDs) caused by high glucose-induced diabetes (Zhang et al., 2020).

Histone β-hydroxybutyrylation is induced by ketogenesis under prolonged fasting (Xie et al., 2016). Ketone bodies including acetoacetic acid, β-hydroxybutyric acid and acetone are intermediate products of liver fatty acid oxidation during starvation, strenuous exercise and diabetes. SIRT3 acts as the de-β-hydroxybutyrylase and selectively catalyzes de-β-hydroxybutyrylation of histones due to its Zn-dependent domain (Zhang X. et al., 2019). Exogenous β-hydroxybutyrate treatment increases H3K9 β-hydroxybutyrylation and activates the expression of MMP-2 and VEGF in diabetic rats (Wu X. et al., 2020). Nevertheless, the role of β-hydroxybutyrylation in tumorigenesis and metastasis remains to be explored.

## Development of Anti-Cancer Therapy By Targeting Cancer Metabolism

Metabolic abnormalities in tumor cells also become the potential targets for the development of anti-cancer drugs. As previously mentioned, some metabolites have anti-cancer activity, including pyruvate, 2DG-6-P, mannose-6-phosphate, and citrate. Most metabolic enzymes that regulate epigenetic modifications are upregulated in tumors and some metabolic enzymes have become targets for cancer treatment (Yu and Li, 2017). There are some drugs targeting glycolysis of tumor cells in preclinical or clinical studies, such as inhibitors for glucose transporters (GLUTs): Phloretin, Fasentin, and lonidamine (LN) (Oudard et al., 2003; Palmieri et al., 2009; Dando et al., 2017), and gossypol, an inhibitor for LDH (Doherty and Cleveland, 2013). In addition, some anti-cancer drugs that target gluconeogenesis have been used in clinical trials. The inhibitor of PEPCK, 3-Mercaptopicolinic acid (MPA) induces tumor cell apoptosis by causing glucose starvation (Wang and Dong, 2019). CM-272 can reverse the Snail-mediated FBP1 expression defects under hypoxic conditions and inhibit the proliferation of hepatocellular carcinoma (HCC) (Barcena-Varela et al., 2019). Dexamethasone promotes gluconeogenesis by enhancing the expression of G6PC and PEPCK, thereby inhibiting the growth of HCC (Wang and Dong, 2019). As we understand more about the role of TCA cycle in tumorigenesis, targeting the TCA cycle to treat cancer has begun to emerge. For example, the IDH2 mutant has become a target for clinical cancer treatment and two drugs have been developed, Enasidenib (AG-221, inhibitors mutant IDH2) and AG-881 (inhibitors of mutant IDH1/2) (Yen et al., 2010). AG-221 is used as a single drug treatment of acute myelogenous leukemia (AML) and solid tumors by reducing intracellular 2-HG (Yen et al., 2010). AG-881 is currently undergoing clinical trials for AML patients with IDH1/2 mutations (Yen et al., 2010). These drugs not only affect cancer metabolism, but also control chromatin modifications. Understanding the relationship between cancer cell metabolism and chromatin modifications not only helps elucidate the mechanism of tumorigenesis, but also facilitates the development of more efficient, accurate, and specific treatment strategies for cancer treatment.

## Discussion

Cancer cells have their specific metabolic pathways and epigenetic modifications that are distinct from normal cells, which contribute to the occurrence and development of tumors. Metabolic reprogram and the concurrent changes in chromatin modifications help tumor cells survive and proliferate in the nutrient-poor environment. Although there is significant progress toward understanding the relationship between cancer metabolism and chromatin modifications, there are still many problems that need to be resolved. First, we and others noticed that some metabolic enzymes and metabolites have both positive and negative effects on cell growth. We have previously reported that pyruvate can inhibit tumor growth by inhibiting histone gene expression (Ma et al., 2019). Pyruvate can also inhibit the activity of HDAC1/3 to trigger apoptosis of colon cancer cells (Thangaraju et al., 2009). However, pyruvate has also been reported to protect cancer cells from DNA damage and oxidative stress under certain circumstance (Tauffenberger et al., 2019). Some metabolic enzymes regulate tumorigenesis in a tissue-dependent manner. PEPCK1 facilitates colorectal cancer proliferation (Yamaguchi et al., 2019); however, it functions as a tumor suppressor in clear cell renal cell carcinoma (ccRCC), liver cancer and hepatocellular carcinoma (HCC) (Liu et al., 2018). Therefore, the anti-tumor drugs developed to target cancer metabolism may also have cell and/or tissue specificity. Secondly, as more metabolic enzymes and metabolites are found to be translocated into the nucleus, elucidating the functions of these metabolic enzymes and metabolites in the nucleus is important for development of anti-cancer therapy.

## Data Availability Statement

The original contributions presented in the study are included in the article/supplementary material, further inquiries can be directed to the corresponding authors.

## Author Contributions

RM, YW, XY, and SL wrote and revised the manuscript. All authors contributed to the article and approved the submitted version.

## Conflict of Interest

The authors declare that the research was conducted in the absence of any commercial or financial relationships that could be construed as a potential conflict of interest.

## References

[B1] Anastasiou D. Poulogiannis G. Asara J. M. Boxer M. B. Jiang J. K. Shen M.. (2011). Inhibition of pyruvate kinase M2 by reactive oxygen species contributes to cellular antioxidant responses. Science 334, 1278–1283. 10.1126/science.1211485 22052977 PMC3471535

[B2] Anderson N. M. Mucka P. Kern J. G. Feng H. (2018). The emerging role and targetability of the TCA cycle in cancer metabolism. Protein Cell 9, 216–237. 10.1007/s13238-017-0451-1 28748451 PMC5818369

[B3] Ballard F. J. Hanson R. W. (1967). The citrate cleavage pathway and lipogenesis in rat adipose tissue: replenishment of oxaloacetate. J. Lipid Res. 8, 73–79. 10.1016/S0022-2275(20)38917-3 14564711

[B4] Bao X. Liu Z. Zhang W. Gladysz K. Fung Y. M. E. Tian G.. (2019). Glutarylation of histone H4 lysine 91 regulates chromatin dynamics. Mol. Cell 76, 660–675 e669. 10.1016/j.molcel.2019.08.018 31542297

[B5] Barcena-Varela M. Caruso S. Llerena S. Alvarez-Sola G. Uriarte I. Latasa M. U.. (2019). Dual targeting of histone methyltransferase G9a and DNA-methyltransferase 1 for the treatment of experimental hepatocellular carcinoma. Hepatology 69, 587–603. 10.1002/hep.30168 30014490

[B6] Bigl M. Jandrig B. Horn L. C. Eschrich K. (2008). Aberrant methylation of human L- and M-fructose 1,6-bisphosphatase genes in cancer. Biochem. Biophys. Res. Commun. 377, 720–724. 10.1016/j.bbrc.2008.10.045 18938139

[B7] Brisson L. Banski P. Sboarina M. Dethier C. Danhier P. Fontenille M. J.. (2016). Lactate dehydrogenase B controls lysosome activity and autophagy in cancer. Cancer Cell 30, 418–431. 10.1016/j.ccell.2016.08.005 27622334

[B8] Brooks G. A. (2020). Lactate as a fulcrum of metabolism. Redox Biol. 35:101454. 10.1016/j.redox.2020.101454 32113910 PMC7284908

[B9] Butler T. P. Grantham F. H. Gullino P. M. (1975). Bulk transfer of fluid in the interstitial compartment of mammary tumors. Cancer Res. 35(11 Pt 1), 3084–3088. 1182701

[B10] Cai Z. Deng Y. Ye J. Zhuo Y. Liu Z. Liang Y.. (2020). Aberrant expression of citrate synthase is linked to disease progression and clinical outcome in prostate cancer. Cancer Manag. Res. 12, 6149–6163. 10.2147/CMAR.S255817 32801864 PMC7398875

[B11] Castonguay Z. Auger C. Thomas S. C. Chahma M. Appanna V. D. (2014). Nuclear lactate dehydrogenase modulates histone modification in human hepatocytes. Biochem. Biophys. Res. Commun. 454, 172–177. 10.1016/j.bbrc.2014.10.071 25450376

[B12] Chaneton B. Gottlieb E. (2012). Rocking cell metabolism: revised functions of the key glycolytic regulator PKM2 in cancer. Trends Biochem. Sci. 37, 309–316. 10.1016/j.tibs.2012.04.003 22626471

[B13] Chang C. Su H. Zhang D. Wang Y. Shen Q. Liu B.. (2015). AMPK-dependent phosphorylation of GAPDH triggers Sirt1 activation and is necessary for autophagy upon glucose starvation. Mol. Cell 60, 930–940. 10.1016/j.molcel.2015.10.037 26626483

[B14] Chang Y. C. Chan Y. C. Chang W. M. Lin Y. F. Yang C. J. Su C. Y.. (2017). Feedback regulation of ALDOA activates the HIF-1alpha/MMP9 axis to promote lung cancer progression. Cancer Lett. 403, 28–36. 10.1016/j.canlet.2017.06.001 28610954

[B15] Chang Y. C. Chiou J. Yang Y. F. Su C. Y. Lin Y. F. Yang C. N.. (2019). Therapeutic targeting of aldolase a interactions inhibits lung cancer metastasis and prolongs survival. Cancer Res. 79, 4754–4766. 10.1158/0008-5472.CAN-18-4080 31358528

[B16] Chen J. Zhang S. Li Y. Tang Z. Kong W. (2014). Hexokinase 2 overexpression promotes the proliferation and survival of laryngeal squamous cell carcinoma. Tumour Biol. 35, 3743–3753. 10.1007/s13277-013-1496-2 24363061

[B17] Chen L. Liu T. Zhou J. Wang Y. Wang X. Di W.. (2014). Citrate synthase expression affects tumor phenotype and drug resistance in human ovarian carcinoma. PLoS ONE 9:e115708. 10.1371/journal.pone.0115708 25545012 PMC4278743

[B18] Chen M. David C. J. Manley J. L. (2012). Concentration-dependent control of pyruvate kinase M mutually exclusive splicing by hnRNP proteins. Nat. Struct. Mol. Biol. 19, 346–354. 10.1038/nsmb.2219 22307054 PMC3698866

[B19] Chen Q. Chen Y. Bian C. Fujiki R. Yu X. (2013). TET2 promotes histone O-GlcNAcylation during gene transcription. Nature 493, 561–564. 10.1038/nature11742 23222540 PMC3684361

[B20] Chen Y. Sprung R. Tang Y. Ball H. Sangras B. Kim S. C.. (2007). Lysine propionylation and butyrylation are novel post-translational modifications in histones. Mol. Cell. Proteomics 6, 812–819. 10.1074/mcp.M700021-MCP200 17267393 PMC2911958

[B21] Cheng A. Zhang P. Wang B. Yang D. Duan X. Jiang Y.. (2019). Aurora-A mediated phosphorylation of LDHB promotes glycolysis and tumor progression by relieving the substrate-inhibition effect. Nat. Commun. 10:5566. 10.1038/s41467-019-13485-8 31804482 PMC6895051

[B22] Chowdhury R. Yeoh K. K. Tian Y. M. Hillringhaus L. Bagg E. A. Rose N. R.. (2011). The oncometabolite 2-hydroxyglutarate inhibits histone lysine demethylases. EMBO Rep. 12, 463–469. 10.1038/embor.2011.43 21460794 PMC3090014

[B23] Chung C. Sweha S. R. Pratt D. Tamrazi B. Panwalkar P. Banda A.. (2020). Integrated metabolic and epigenomic reprograming by H3K27M mutations in diffuse intrinsic pontine gliomas. Cancer Cell 38, 334–349 e339. 10.1016/j.ccell.2020.07.008 32795401 PMC7494613

[B24] Colell A. Ricci J. E. Tait S. Milasta S. Maurer U. Bouchier-Hayes L.. (2007). GAPDH and autophagy preserve survival after apoptotic cytochrome c release in the absence of caspase activation. Cell 129, 983–997. 10.1016/j.cell.2007.03.045 17540177

[B25] Dall'olio F. Trinchera M. (2017). Epigenetic bases of aberrant glycosylation in cancer. Int. J. Mol. Sci. 18:998. 10.3390/ijms18050998 28481247 PMC5454911

[B26] Dando I. Pacchiana R. Pozza E. D. Cataldo I. Bruno S. Conti P.. (2017). UCP2 inhibition induces ROS/Akt/mTOR axis: role of GAPDH nuclear translocation in genipin/everolimus anticancer synergism. Free Radic. Biol. Med. 113, 176–189. 10.1016/j.freeradbiomed.2017.09.022 28962872

[B27] Dang C. V. Semenza G. L. (1999). Oncogenic alterations of metabolism. Trends Biochem. Sci. 24, 68–72. 10.1016/S0968-0004(98)01344-9 10098401

[B28] Dang L. Yen K. Attar E. C. (2016). IDH mutations in cancer and progress toward development of targeted therapeutics. Ann. Oncol. 27, 599–608. 10.1093/annonc/mdw013 27005468

[B29] De Saedeleer C. J. Copetti T. Porporato P. E. Verrax J. Feron O. Sonveaux P. (2012). Lactate activates HIF-1 in oxidative but not in Warburg-phenotype human tumor cells. PLoS ONE 7:e46571. 10.1371/journal.pone.0046571 23082126 PMC3474765

[B30] Dehennaut V. Leprince D. Lefebvre T. (2014). O-GlcNAcylation, an epigenetic mark. Focus on the histone code, TET family proteins, and polycomb group proteins. Front. Endocrinol. 5:155. 10.3389/fendo.2014.00155 25309514 PMC4176146

[B31] Doherty J. R. Cleveland J. L. (2013). Targeting lactate metabolism for cancer therapeutics. J. Clin. Invest. 123, 3685–3692. 10.1172/JCI69741 23999443 PMC3754272

[B32] Du J. Zhou Y. Su X. Yu J. J. Khan S. Jiang H.. (2011). Sirt5 is a NAD-dependent protein lysine demalonylase and desuccinylase. Science 334, 806–809. 10.1126/science.1207861 22076378 PMC3217313

[B33] Du S. Guan Z. Hao L. Song Y. Wang L. Gong L.. (2014). Fructose-bisphosphate aldolase a is a potential metastasis-associated marker of lung squamous cell carcinoma and promotes lung cell tumorigenesis and migration. PLoS ONE 9:e85804. 10.1371/journal.pone.0085804 24465716 PMC3900443

[B34] Fan J. Hitosugi T. Chung T. W. Xie J. Ge Q. Gu T. L.. (2011). Tyrosine phosphorylation of lactate dehydrogenase A is important for NADH/NAD(+) redox homeostasis in cancer cells. Mol. Cell. Biol. 31, 4938–4950. 10.1128/MCB.06120-11 21969607 PMC3233034

[B35] Fan T. Sun G. Sun X. Zhao L. Zhong R. Peng Y. (2019). Tumor energy metabolism and potential of 3-bromopyruvate as an inhibitor of aerobic glycolysis: implications in tumor treatment. Cancers 11:317. 10.3390/cancers11030317 30845728 PMC6468516

[B36] Fantin V. R. St-Pierre J. Leder P. (2006). Attenuation of LDH-A expression uncovers a link between glycolysis, mitochondrial physiology, and tumor maintenance. Cancer Cell 9, 425–434. 10.1016/j.ccr.2006.04.023 16766262

[B37] Feng Y. Xiong Y. Qiao T. Li X. Jia L. Han Y. (2018). Lactate dehydrogenase A: a key player in carcinogenesis and potential target in cancer therapy. Cancer Med. 7, 6124–6136. 10.1002/cam4.1820 30403008 PMC6308051

[B38] Fortier S. Labelle D. Sina A. Moreau R. Annabi B. (2008). Silencing of the MT1-MMP/ G6PT axis suppresses calcium mobilization by sphingosine-1-phosphate in glioblastoma cells. FEBS Lett. 582, 799–804. 10.1016/j.febslet.2008.01.061 18267120

[B39] Fruman D. A. Rommel C. (2014). PI3K and cancer: lessons, challenges and opportunities. Nat. Rev. Drug Discov. 13, 140–156. 10.1038/nrd4204 24481312 PMC3994981

[B40] Fu H. Gao H. Qi X. Zhao L. Wu D. Bai Y.. (2018). Aldolase A promotes proliferation and G1/S transition via the EGFR/MAPK pathway in non-small cell lung cancer. Cancer Commun. 38:18. 10.1186/s40880-018-0290-3 29764507 PMC5993145

[B41] Fu L. N. Wang Y. Q. Tan J. Xu J. Gao Q. Y. Chen Y. X.. (2018). Role of JMJD2B in colon cancer cell survival under glucose-deprived conditions and the underlying mechanisms. Oncogene 37, 389–402. 10.1038/onc.2017.345 28945223

[B42] Funasaka T. Hogan V. Raz A. (2009). Phosphoglucose isomerase/autocrine motility factor mediates epithelial and mesenchymal phenotype conversions in breast cancer. Cancer Res. 69, 5349–5356. 10.1158/0008-5472.CAN-09-0488 19531650 PMC2875197

[B43] Funasaka T. Yanagawa T. Hogan V. Raz A. (2005). Regulation of phosphoglucose isomerase/autocrine motility factor expression by hypoxia. FASEB J. 19, 1422–1430. 10.1096/fj.05-3699com 16126909

[B44] Gonzalez P. S. O'prey J. Cardaci S. Barthet V. J.A. Sakamaki J. I. Beaumatin F.. (2018). Mannose impairs tumour growth and enhances chemotherapy. Nature 563, 719–723. 10.1038/s41586-018-0729-3 30464341

[B45] Gras D. Roze E. Caillet S. Meneret A. Doummar D. Billette De Villemeur T.. (2014). GLUT1 deficiency syndrome: an update. Rev. Neurol. 170, 91–99. 10.1016/j.neurol.2013.09.005 24269118

[B46] Guo T. Chen T. Gu C. Li B. Xu C. (2015). Genetic and molecular analyses reveal G6PC as a key element connecting glucose metabolism and cell cycle control in ovarian cancer. Tumour Biol. 36, 7649–7658. 10.1007/s13277-015-3463-6 25926381

[B47] Gutteridge R. E. Singh C. K. Ndiaye M. A. Ahmad N. (2017). Targeted knockdown of polo-like kinase 1 alters metabolic regulation in melanoma. Cancer Lett. 394, 13–21. 10.1016/j.canlet.2017.02.013 28235541 PMC5415376

[B48] Haga A. Funasaka T. Niinaka Y. Raz A. Nagase H. (2003). Autocrine motility factor signaling induces tumor apoptotic resistance by regulations Apaf-1 and Caspase-9 apoptosome expression. Int. J. Cancer 107, 707–714. 10.1002/ijc.11449 14566819

[B49] Han Z. Wu H. Kim S. Yang X. Li Q. Huang H.. (2018). Revealing the protein propionylation activity of the histone acetyltransferase MOF (males absent on the first). J. Biol. Chem. 293, 3410–3420. 10.1074/jbc.RA117.000529 29321206 PMC5836141

[B50] Hanahan D. Weinberg R. A. (2011). Hallmarks of cancer: the next generation. Cell 144, 646–674. 10.1016/j.cell.2011.02.013 21376230

[B51] Hara M. R. Agrawal N. Kim S. F. Cascio M. B. Fujimuro M. Ozeki Y.. (2005). S-nitrosylated GAPDH initiates apoptotic cell death by nuclear translocation following Siah1 binding. Nat. Cell Biol. 7, 665–674. 10.1038/ncb1268 15951807

[B52] He C. L. Bian Y. Y. Xue Y. Liu Z. X. Zhou K. Q. Yao C. F.. (2016). Pyruvate Kinase M2 Activates mTORC1 by Phosphorylating AKT1S1. Sci. Rep. 6:21524. 10.1038/srep21524 26876154 PMC4753445

[B53] Hirata H. Sugimachi K. Komatsu H. Ueda M. Masuda T. Uchi R.. (2016). Decreased expression of fructose-1,6-bisphosphatase associates with glucose metabolism and tumor progression in hepatocellular carcinoma. Cancer Res. 76, 3265–3276. 10.1158/0008-5472.CAN-15-2601 27197151

[B54] Hitosugi T. Kang S. Vander Heiden M. G. Chung T. W. Elf S. Lythgoe K.. (2009). Tyrosine phosphorylation inhibits PKM2 to promote the Warburg effect and tumor growth. Sci. Signal. 2:ra73. 10.1126/scisignal.2000431 19920251 PMC2812789

[B55] Hu X. Chao M. Wu H. (2017). Central role of lactate and proton in cancer cell resistance to glucose deprivation and its clinical translation. Signal. Transduct. Target Ther. 2:16047. 10.1038/sigtrans.2016.47 29263910 PMC5661620

[B56] Hua Y. Liang C. Zhu J. Miao C. Yu Y. Xu A.. (2017). Expression of lactate dehydrogenase C correlates with poor prognosis in renal cell carcinoma. Tumour Biol. 39:1010428317695968. 10.1177/1010428317695968 28351304

[B57] Huang L. Wang C. Xu H. Peng G. (2020). Targeting citrate as a novel therapeutic strategy in cancer treatment. Biochim. Biophys. Acta Rev. Cancer 1873:188332. 10.1016/j.bbcan.2019.188332 31751601

[B58] Ishiguro T. Tanabe K. Kobayashi Y. Mizumoto S. Kanai M. Kawashima S. A. (2018). Malonylation of histone H2A at lysine 119 inhibits Bub1-dependent H2A phosphorylation and chromosomal localization of shugoshin proteins. Sci. Rep. 8:7671. 10.1038/s41s598-018-26114-z 29769606 PMC5956101

[B59] Israelsen W. J. Vander Heiden M. G. (2015). Pyruvate kinase: function, regulation and role in cancer. Semin. Cell Dev. Biol. 43, 43–51. 10.1016/j.semcdb.2015.08.004 26277545 PMC4662905

[B60] Ji S. Zhang B. Liu J. Qin Y. Liang C. Shi S.. (2016). ALDOA functions as an oncogene in the highly metastatic pancreatic cancer. Cancer Lett. 374, 127–135. 10.1016/j.canlet.2016.01.054 26854714

[B61] Ji Y. Yang C. Tang Z. Yang Y. Tian Y. Yao H.. (2017). Adenylate kinase hCINAP determines self-renewal of colorectal cancer stem cells by facilitating LDHA phosphorylation. Nat. Commun. 8:15308. 10.1038/ncomms16000 28516914 PMC5454382

[B62] Jia G. Fu Y. Zhao X. Dai Q. Zheng G. Yang Y.. (2011). N6-methyladenosine in nuclear RNA is a major substrate of the obesity-associated FTO. Nat. Chem. Biol. 7, 885–887. 10.1038/nchembio.687 22002720 PMC3218240

[B63] Jiang S. Zhang L. F. Zhang H. W. Hu S. Lu M. H. Liang S.. (2012). A novel miR-155/miR-143 cascade controls glycolysis by regulating hexokinase 2 in breast cancer cells. EMBO J. 31, 1985–1998. 10.1038/emboj.2012.45 22354042 PMC3343331

[B64] Jitrapakdee S. Vidal-Puig A. Wallace J. C. (2006). Anaplerotic roles of pyruvate carboxylase in mammalian tissues. Cell. Mol. Life Sci. 63, 843–854. 10.1007/s00018-005-5410-y 16505973 PMC11136034

[B65] Kajimoto K. Terada H. Baba Y. Shinohara Y. (2005). Essential role of citrate export from mitochondria at early differentiation stage of 3T3-L1 cells for their effective differentiation into fat cells, as revealed by studies using specific inhibitors of mitochondrial di- and tricarboxylate carriers. Mol. Genet. Metab. 85, 46–53. 10.1016/j.ymgme.2005.01.006 15862280

[B66] Kawai K. Uemura M. Munakata K. Takahashi H. Haraguchi N. Nishimura J.. (2017). Fructose-bisphosphate aldolase A is a key regulator of hypoxic adaptation in colorectal cancer cells and involved in treatment resistance and poor prognosis. Int. J. Oncol. 50, 525–534. 10.3892/ijo.2016.3814 28000858

[B67] Kebede A. F. Nieborak A. Shahidian L. Z. Le Gras S. Richter F. Gomez D. A.. (2017). Histone propionylation is a mark of active chromatin. Nat. Struct. Mol. Biol. 24, 1048–1056. 10.1038/nsmb.3490 29058708

[B68] Kim J. Y. Banerjee T. Vinckevicius A. Luo Q. Parker J. B. Baker M. R.. (2014). A role for WDR5 in integrating threonine 11 phosphorylation to lysine 4 methylation on histone H3 during androgen signaling and in prostate cancer. Mol. Cell 54, 613–625. 10.1016/j.molcel.2014.03.043 24793694 PMC4075454

[B69] Kollenstart L. De Groot A. J.L. Janssen G. M.C. Cheng X. Vreeken K. Martino F.. (2019). Gcn5 and Esa1 function as histone crotonyltransferases to regulate crotonylation-dependent transcription. J. Biol. Chem. 294, 20122–20134. 10.1074/jbc.RA119.010302 31699900 PMC6937567

[B70] Kornberg M. D. Sen N. Hara M. R. Juluri K. R. Nguyen J. V. Snowman A. M.. (2010). GAPDH mediates nitrosylation of nuclear proteins. Nat. Cell Biol. 12, 1094–1100. 10.1038/ncb2114 20972425 PMC2972384

[B71] Kwee S. A. Hernandez B. Chan O. Wong L. (2012). Choline kinase alpha and hexokinase-2 protein expression in hepatocellular carcinoma: association with survival. PLoS ONE 7:e46591. 10.1371/journal.pone.0046591 23071593 PMC3465336

[B72] Lang L. Chemmalakuzhy R. Shay C. Teng Y. (2019). PFKP signaling at a glance: an emerging mediator of cancer cell metabolism. Adv. Exp. Med. Biol. 1134, 243–258. 10.1007/978-3-030-12668-1_13 30919341

[B73] Le A. Cooper C. R. Gouw A. M. Dinavahi R. Maitra A. Deck L. M.. (2010). Inhibition of lactate dehydrogenase A induces oxidative stress and inhibits tumor progression. Proc. Natl. Acad. Sci. U.S.A. 107, 2037–2042. 10.1073/pnas.0914433107 20133848 PMC2836706

[B74] Lee J. H. Liu R. Li J. Wang Y. Tan L. Li X. J.. (2018). EGFR-phosphorylated platelet isoform of phosphofructokinase 1 promotes PI3K activation. Mol. Cell 70, 197–210 e197. 10.1016/j.molcel.2018.03.018 29677490 PMC6114939

[B75] Lee J. H. Liu R. Li J. Zhang C. Wang Y. Cai Q.. (2017). Stabilization of phosphofructokinase 1 platelet isoform by AKT promotes tumorigenesis. Nat. Commun. 8:949. 10.1038/s41467-017-00906-9 29038421 PMC5643558

[B76] Lee M. N. Ha S. H. Kim J. Koh A. Lee C. S. Kim J. H.. (2009). Glycolytic flux signals to mTOR through glyceraldehyde-3-phosphate dehydrogenase-mediated regulation of Rheb. Mol. Cell. Biol. 29, 3991–4001. 10.1128/MCB.00165-09 19451232 PMC2704738

[B77] Li H. Wang J. Xu H. Xing R. Pan Y. Li W.. (2013). Decreased fructose-1,6-bisphosphatase-2 expression promotes glycolysis and growth in gastric cancer cells. Mol. Cancer 12:110. 10.1186/1476-4598-12-110 24063558 PMC3849177

[B78] Li Q. Li Y. Xu J. Wang S. Xu Y. Li X.. (2017). Aldolase B overexpression is associated with poor prognosis and promotes tumor progression by epithelial-mesenchymal transition in colorectal adenocarcinoma. Cell. Physiol. Biochem. 42, 397–406. 10.1159/000477484 28558381

[B79] Li Q. Zhang D. Chen X. He L. Li T. Xu X.. (2015). Nuclear PKM2 contributes to gefitinib resistance via upregulation of STAT3 activation in colorectal cancer. Sci. Rep 5, 16082. 10.1038/srep16082 26542452 PMC4635355

[B80] Li S. Swanson S. K. Gogol M. Florens L. Washburn M. P. Workman J. L.. (2015). Serine and SAM responsive complex SESAME regulates histone modification crosstalk by sensing cellular metabolism. Mol. Cell 60, 408–421. 10.1016/j.molcel.2015.09.024 26527276

[B81] Li Y. Luo S. Ma R. Liu J. Xu P. Zhang H.. (2015). Upregulation of cytosolic phosphoenolpyruvate carboxykinase is a critical metabolic event in melanoma cells that repopulate tumors. Cancer Res. 75, 1191–1196. 10.1158/0008-5472.CAN-14-2615 25712344 PMC4629827

[B82] Liang J. Cao R. Wang X. Zhang Y. Wang P. Gao H.. (2017). Mitochondrial PKM2 regulates oxidative stress-induced apoptosis by stabilizing Bcl2. Cell Res. 27, 329–351. 10.1038/cr.2016.159 28035139 PMC5339831

[B83] Lincet H. Icard P. (2015). How do glycolytic enzymes favour cancer cell proliferation by nonmetabolic functions? Oncogene 34, 3751–3759. 10.1038/onc.2014.320 25263450

[B84] Linden M. Gellerfors P. Nelson B. D. (1982). Pore protein and the hexokinase-binding protein from the outer membrane of rat liver mitochondria are identical. FEBS Lett. 141, 189–192. 10.1016/0014-5793(82)80044-6 6178620

[B85] Linke C. Wosle M. Harder A. (2020). Anti-cancer agent 3-bromopyruvate reduces growth of MPNST and inhibits metabolic pathways in a representative *in-vitro* model. BMC Cancer 20:896. 10.1186/s12885-020-07397-w 32948135 PMC7501688

[B86] Liu B. Lin Y. Darwanto A. Song X. Xu G. Zhang K. (2009). Identification and characterization of propionylation at histone H3 lysine 23 in mammalian cells. J. Biol. Chem. 284, 32288–32295. 10.1074/jbc.M109.045856 19801601 PMC2781642

[B87] Liu H. E. Shi H. H. Luo X. J. (2020). Upregulated long noncoding RNA UCA1 enhances warburg effect via miR-203/HK2 axis in esophagal cancer. J. Oncol. 2020:8847687. 10.1155/2020/8847687 33204264 PMC7657677

[B88] Liu J. Peng Y. Shi L. Wan L. Inuzuka H. Long J.. (2021). Skp2 dictates cell cycle-dependent metabolic oscillation between glycolysis and TCA cycle. Cell Res. 31, 80–93. 10.1038/s41422-020-0372-z 32669607 PMC7852548

[B89] Liu M. X. Jin L. Sun S. J. Liu P. Feng X. Cheng Z. L.. (2018). Metabolic reprogramming by PCK1 promotes TCA cataplerosis, oxidative stress and apoptosis in liver cancer cells and suppresses hepatocellular carcinoma. Oncogene 37, 1637–1653. 10.1038/s41388-017-0070-6 29335519 PMC5860930

[B90] Liu Q. Huo Y. Zheng H. Zhao J. Jia L. Wang P. (2019). Ethyl pyruvate suppresses the growth, invasion and migration and induces the apoptosis of nonsmall cell lung cancer cells via the HMGB1/RAGE axis and the NFkappaB/STAT3 pathway. Oncol. Rep. 42, 817–825. 10.3892/or.2019.7176 31173265

[B91] Liu S. Yu H. Liu Y. Liu X. Zhang Y. Bu C.. (2017). Chromodomain protein CDYL acts as a crotonyl-CoA hydratase to regulate histone crotonylation and spermatogenesis. Mol. Cell 67, 853–866 e855. 10.1016/j.molcel.2017.07.011 28803779

[B92] Liu X. S. Little J. B. Yuan Z. M. (2015). Glycolytic metabolism influences global chromatin structure. Oncotarget 6, 4214–4225. 10.18632/oncotarget.2929 25784656 PMC4414184

[B93] Liu Y. Li M. Fan M. Song Y. Yu H. Zhi X.. (2019). Chromodomain Y-like protein-mediated histone crotonylation regulates stress-induced depressive behaviors. Biol. Psychiatry 85, 635–649. 10.1016/j.biopsych.2018.11.025 30665597

[B94] Lo P. W. Shie J. J. Chen C. H. Wu C. Y. Hsu T. L. Wong C. H. (2018). O-GlcNAcylation regulates the stability and enzymatic activity of the histone methyltransferase EZH2. Proc. Natl. Acad. Sci. U.S.A. 115, 7302–7307. 10.1073/pnas.1801850115 29941599 PMC6048490

[B95] Lord-Dufour S. Copland I. B. Levros L. C. Jr. Post M. Das A. Khosla C.. (2009). Evidence for transcriptional regulation of the glucose-6-phosphate transporter by HIF-1alpha: targeting G6PT with mumbaistatin analogs in hypoxic mesenchymal stromal cells. Stem Cells 27, 489–497. 10.1634/stemcells.2008-0855 19074414 PMC2728688

[B96] Luo W. Hu H. Chang R. Zhong J. Knabel M. O'meally R.. (2011). Pyruvate kinase M2 is a PHD3-stimulated coactivator for hypoxia-inducible factor 1. Cell 145, 732–744. 10.1016/j.cell.2011.03.054 21620138 PMC3130564

[B97] Lv L. Li D. Zhao D. Lin R. Chu Y. Zhang H.. (2011). Acetylation targets the M2 isoform of pyruvate kinase for degradation through chaperone-mediated autophagy and promotes tumor growth. Mol. Cell 42, 719–730. 10.1016/j.molcel.2011.04.025 21700219 PMC4879880

[B98] Lv L. Xu Y. P. Zhao D. Li F. L. Wang W. Sasaki N.. (2013). Mitogenic and oncogenic stimulation of K433 acetylation promotes PKM2 protein kinase activity and nuclear localization. Mol. Cell 52, 340–352. 10.1016/j.molcel.2013.09.004 24120661 PMC4183148

[B99] Ma R. Wu Y. Zhai Y. Hu B. Ma W. Yang W.. (2019). Exogenous pyruvate represses histone gene expression and inhibits cancer cell proliferation via the NAMPT-NAD+-SIRT1 pathway. Nucleic Acids Res. 47, 11132–11150. 10.1093/nar/gkz864 31598701 PMC6868375

[B100] MacPherson S. Horkoff M. Gravel C. Hoffmann T. Zuber J. Lum J. J. (2017). STAT3 regulation of citrate synthase is essential during the initiation of lymphocyte cell growth. Cell Rep. 19, 910–918. 10.1016/j.celrep.2017.04.012 28467904

[B101] Mazurek S. (2011). Pyruvate kinase type M2: a key regulator of the metabolic budget system in tumor cells. Int. J. Biochem. Cell Biol. 43, 969–980. 10.1016/j.biocel.2010.02.005 20156581

[B102] Mcbrian M. A. Behbahan I. S. Ferrari R. Su T. Huang T. W. Li K.. (2013). Histone acetylation regulates intracellular pH. Mol. Cell 49, 310–321. 10.1016/j.molcel.2012.10.025 23201122 PMC3893119

[B103] Mendez-Lucas A. Hyrossova P. Novellasdemunt L. Vinals F. Perales J. C. (2014). Mitochondrial phosphoenolpyruvate carboxykinase (PEPCK-M) is a pro-survival, endoplasmic reticulum (ER) stress response gene involved in tumor cell adaptation to nutrient availability. J. Biol. Chem. 289, 22090–22102. 10.1074/jbc.M114.566927 24973213 PMC4139223

[B104] Miao P. Sheng S. Sun X. Liu J. Huang G. (2013). Lactate dehydrogenase A in cancer: a promising target for diagnosis and therapy. IUBMB Life 65, 904–910. 10.1002/iub.1216 24265197

[B105] Min J. W. Kim K. I. Kim H. A. Kim E. K. Noh W. C. Jeon H. B.. (2013). INPP4B-mediated tumor resistance is associated with modulation of glucose metabolism via hexokinase 2 regulation in laryngeal cancer cells. Biochem. Biophys. Res. Commun. 440, 137–142. 10.1016/j.bbrc.2013.09.041 24051093

[B106] Mohammad G. H. Vassileva V. Acedo P. Olde Damink S. W.M. Malago M. Dhar D. K.. (2019). Targeting pyruvate kinase M2 and lactate dehydrogenase a is an effective combination strategy for the treatment of pancreatic cancer. Cancers 11:1372. 10.3390/cancers11091372 31527446 PMC6770573

[B107] Moore L. E. Jaeger E. Nickerson M. L. Brennan P. De Vries S. Roy R.. (2012). Genomic copy number alterations in clear cell renal carcinoma: associations with case characteristics and mechanisms of VHL gene inactivation. Oncogenesis 1:e14. 10.1038/oncsis.2012.14 23552698 PMC3412648

[B108] Mor I. Cheung E. C. Vousden K. H. (2011). Control of glycolysis through regulation of PFK1: old friends and recent additions. Cold Spring Harb. Symp. Quant. Biol. 76, 211–216. 10.1101/sqb.2011.76.010868 22096029

[B109] Morita M. Sato T. Nomura M. Sakamoto Y. Inoue Y. Tanaka R.. (2018). PKM1 confers metabolic advantages and promotes cell-autonomous tumor cell growth. Cancer Cell 33, 355–367 e357. 10.1016/j.ccell.2018.02.004 29533781

[B110] Nishikawa K. Iwamoto Y. Kobayashi Y. Katsuoka F. Kawaguchi S. Tsujita T.. (2015). DNA methyltransferase 3a regulates osteoclast differentiation by coupling to an S-adenosylmethionine-producing metabolic pathway. Nat. Med. 21, 281–287. 10.1038/nm.3774 25706873

[B111] Oudard S. Carpentier A. Banu E. Fauchon F. Celerier D. Poupon M. F.. (2003). Phase II study of lonidamine and diazepam in the treatment of recurrent glioblastoma multiforme. J. Neurooncol. 63, 81–86. 10.1023/A:1023756707900 12814259

[B112] Palmieri D. Fitzgerald D. Shreeve S. M. Hua E. Bronder J. L. Weil R. J.. (2009). Analyses of resected human brain metastases of breast cancer reveal the association between up-regulation of hexokinase 2 and poor prognosis. Mol. Cancer Res. 7, 1438–1445. 10.1158/1541-7786.MCR-09-0234 19723875 PMC2746883

[B113] Pavlova N. N. Thompson C. B. (2016). The emerging hallmarks of cancer metabolism. Cell Metab. 23, 27–47. 10.1016/j.cmet.2015.12.006 26771115 PMC4715268

[B114] Peschiaroli A. Giacobbe A. Formosa A. Markert E. K. Bongiorno-Borbone L. Levine A. J.. (2013). miR-143 regulates hexokinase 2 expression in cancer cells. Oncogene 32, 797–802. 10.1038/onc.2012.100 22469988

[B115] Phannasil P. Ansari I. H. El Azzouny M. Longacre M. J. Rattanapornsompong K. Burant C. F.. (2017). Mass spectrometry analysis shows the biosynthetic pathways supported by pyruvate carboxylase in highly invasive breast cancer cells. Biochim. Biophys. Acta Mol. Basis Dis. 1863, 537–551. 10.1016/j.bbadis.2016.11.021 27890529 PMC5243144

[B116] Phannasil P. Thuwajit C. Warnnissorn M. Wallace J. C. Macdonald M. J. Jitrapakdee S. (2015). Pyruvate carboxylase is up-regulated in breast cancer and essential to support growth and invasion of MDA-MB-231 cells. PLoS ONE 10:e0129848. 10.1371/journal.pone.0129848 26070193 PMC4467472

[B117] Pusapati R. V. Daemen A. Wilson C. Sandoval W. Gao M. Haley B.. (2016). mTORC1-dependent metabolic reprogramming underlies escape from glycolysis addiction in cancer cells. Cancer Cell 29, 548–562. 10.1016/j.ccell.2016.02.018 27052953

[B118] Qing Y. Dong L. Gao L. Li C. Li Y. Han L.. (2021). R-2-hydroxyglutarate attenuates aerobic glycolysis in leukemia by targeting the FTO/m(6)A/PFKP/LDHB axis. Mol Cell. 81, 922–939.e9. 10.1016/j.molcel.2020.12.026 33434505 PMC7935770

[B119] Rabinowitz J. D. Enerback S. (2020). Lactate: the ugly duckling of energy metabolism. Nat. Metab. 2, 566–571. 10.1038/s42255-020-0243-4 32694798 PMC7983055

[B120] Reshef L. Olswang Y. Cassuto H. Blum B. Croniger C. M. Kalhan S. C.. (2003). Glyceroneogenesis and the triglyceride/fatty acid cycle. J. Biol. Chem. 278, 30413–30416. 10.1074/jbc.R300017200 12788931

[B121] Ringel A. E. Ryznar R. Picariello H. Huang K. L. Lazarus A. G. Holmes S. G. (2013). Yeast Tdh3 (glyceraldehyde 3-phosphate dehydrogenase) is a Sir2-interacting factor that regulates transcriptional silencing and rDNA recombination. PLoS Genet. 9:e1003871. 10.1371/journal.pgen.1003871 24146631 PMC3798266

[B122] Roberts D. J. Tan-Sah V. P. Ding E. Y. Smith J. M. Miyamoto S. (2014). Hexokinase-II positively regulates glucose starvation-induced autophagy through TORC1 inhibition. Mol. Cell 53, 521–533. 10.1016/j.molcel.2013.12.019 24462113 PMC3943874

[B123] Sabari B. R. Zhang D. Allis C. D. Zhao Y. (2017). Metabolic regulation of gene expression through histone acylations. Nat. Rev. Mol. Cell Biol. 18, 90–101. 10.1038/nrm.2016.140 27924077 PMC5320945

[B124] Sajnani K. Islam F. Smith R. A. Gopalan V. Lam A. K. (2017). Genetic alterations in Krebs cycle and its impact on cancer pathogenesis. Biochimie 135, 164–172. 10.1016/j.biochi.2017.02.008 28219702

[B125] Sakabe K. Hart G. W. (2010). O-GlcNAc transferase regulates mitotic chromatin dynamics. J. Biol. Chem. 285, 34460–34468. 10.1074/jbc.M110.158170 20805223 PMC2966060

[B126] San-Millan I. Julian C. G. Matarazzo C. Martinez J. Brooks G. A. (2019). Is lactate an oncometabolite? Evidence supporting a role for lactate in the regulation of transcriptional activity of cancer-related genes in MCF7 breast cancer cells. Front. Oncol. 9:1536. 10.3389/fonc.2019.01536 32010625 PMC6971189

[B127] Sen N. Hara M. R. Kornberg M. D. Cascio M. B. Bae B. I. Shahani N.. (2008). Nitric oxide-induced nuclear GAPDH activates p300/CBP and mediates apoptosis. Nat. Cell Biol. 10, 866–873. 10.1038/ncb1747 18552833 PMC2689382

[B128] Sheng S. L. Liu J. J. Dai Y. H. Sun X. G. Xiong X. P. Huang G. (2012). Knockdown of lactate dehydrogenase A suppresses tumor growth and metastasis of human hepatocellular carcinoma. FEBS J. 279, 3898–3910. 10.1111/j.1742-4658.2012.08748.x 22897481

[B129] Shi L. An S. Liu Y. Liu J. Wang F. (2020). PCK1 regulates glycolysis and tumor progression in clear cell renal cell carcinoma through LDHA. Onco. Targets Ther. 13, 2613–2627. 10.2147/OTT.S241717 32280238 PMC7125947

[B130] Shi L. Yan H. An S. Shen M. Jia W. Zhang R.. (2019). SIRT5-mediated deacetylation of LDHB promotes autophagy and tumorigenesis in colorectal cancer. Mol. Oncol. 13, 358–375. 10.1002/1878-0261.12408 30443978 PMC6360364

[B131] Shimizu T. Inoue K. Hachiya H. Shibuya N. Shimoda M. Kubota K. (2014). Frequent alteration of the protein synthesis of enzymes for glucose metabolism in hepatocellular carcinomas. J. Gastroenterol. 49, 1324–1332. 10.1007/s00535-013-0895-x 24203292 PMC4156784

[B132] Shin D. Lee J. You J. H. Kim D. Roh J. L. (2020). Dihydrolipoamide dehydrogenase regulates cystine deprivation-induced ferroptosis in head and neck cancer. Redox Biol. 30:101418. 10.1016/j.redox.2019.101418 31931284 PMC6957841

[B133] Shyh-Chang N. Locasale J. W. Lyssiotis C. A. Zheng Y. Teo R. Y. Ratanasirintrawoot S.. (2013). Influence of threonine metabolism on S-adenosylmethionine and histone methylation. Science 339, 222–226. 10.1126/science.1226603 23118012 PMC3652341

[B134] Sims J. Bruschi C. V. Bertin C. West N. Breitenbach M. Schroeder S.. (2016). High reactive oxygen species levels are detected at the end of the chronological life span of translocant yeast cells. Mol. Genet. Genomics 291, 423–435. 10.1007/s00438-015-1120-9 26423068

[B135] Sivanand S. Viney I. Wellen K. E. (2018). Spatiotemporal control of acetyl-CoA metabolism in chromatin regulation. Trends Biochem. Sci. 43, 61–74. 10.1016/j.tibs.2017.11.004 29174173 PMC5741483

[B136] Slane B. G. Aykin-Burns N. Smith B. J. Kalen A. L. Goswami P. C. Domann F. E.. (2006). Mutation of succinate dehydrogenase subunit C results in increased O2.-, oxidative stress, and genomic instability. Cancer Res. 66, 7615–7620. 10.1158/0008-5472.CAN-06-0833 16885361

[B137] Slawson C. Lakshmanan T. Knapp S. Hart G. W. (2008). A mitotic GlcNAcylation/phosphorylation signaling complex alters the posttranslational state of the cytoskeletal protein vimentin. Mol. Biol. Cell 19, 4130–4140. 10.1091/mbc.e07-11-1146 18653473 PMC2555957

[B138] Su R. Dong L. Li C. Nachtergaele S. Wunderlich M. Qing Y.. (2018). R-2HG exhibits anti-tumor activity by targeting FTO/m(6)A/MYC/CEBPA signaling. Cell 172, 90–105 e123. 10.1016/j.cell.2017.11.031 29249359 PMC5766423

[B139] Tao Q. F. Yuan S. X. Yang F. Yang S. Yang Y. Yuan J. H.. (2015). Aldolase B inhibits metastasis through Ten-Eleven Translocation 1 and serves as a prognostic biomarker in hepatocellular carcinoma. Mol. Cancer 14:170. 10.1186/s12943-015-0437-7 26376879 PMC4574028

[B140] Tauffenberger A. Fiumelli H. Almustafa S. Magistretti P. J. (2019). Lactate and pyruvate promote oxidative stress resistance through hormetic ROS signaling. Cell Death Dis. 10:653. 10.1038/s41419-019-1877-6 31506428 PMC6737085

[B141] Thangaraju M. Carswell K. N. Prasad P. D. Ganapathy V. (2009). Colon cancer cells maintain low levels of pyruvate to avoid cell death caused by inhibition of HDAC1/HDAC3. Biochem. J. 417, 379–389. 10.1042/BJ20081132 18789002

[B142] Tosato V. Gruning N. M. Breitenbach M. Arnak R. Ralser M. Bruschi C. V. (2012). Warburg effect and translocation-induced genomic instability: two yeast models for cancer cells. Front. Oncol. 2:212. 10.3389/fonc.2012.00212 23346549 PMC3548335

[B143] Tran K. A. Dillingham C. M. Sridharan R. (2019). The role of alpha-ketoglutarate-dependent proteins in pluripotency acquisition and maintenance. J. Biol. Chem. 294, 5408–5419. 10.1074/jbc.TM118.000831 30181211 PMC6462505

[B144] Tristan C. Shahani N. Sedlak T. W. Sawa A. (2011). The diverse functions of GAPDH: views from different subcellular compartments. Cell. Signal. 23, 317–323. 10.1016/j.cellsig.2010.08.003 20727968 PMC3084531

[B145] Tsutsumi S. Fukasawa T. Yamauchi H. Kato T. Kigure W. Morita H.. (2009). Phosphoglucose isomerase enhances colorectal cancer metastasis. Int. J. Oncol. 35, 1117–1121. 10.3892/ijo_00000427 19787266

[B146] Tsutsumi S. Yanagawa T. Shimura T. Kuwano H. Raz A. (2004). Autocrine motility factor signaling enhances pancreatic cancer metastasis. Clin. Cancer Res. 10, 7775–7784. 10.1158/1078-0432.CCR-04-1015 15570012

[B147] Tuo L. Xiang J. Pan X. Gao Q. Zhang G. Yang Y.. (2018). PCK1 Downregulation promotes TXNRD1 expression and hepatoma cell growth via the Nrf2/Keap1 pathway. Front. Oncol. 8:611. 10.3389/fonc.2018.00611 30619751 PMC6304441

[B148] Tuo L. Xiang J. Pan X. Hu J. Tang H. Liang L.. (2019). PCK1 negatively regulates cell cycle progression and hepatoma cell proliferation via the AMPK/p27(Kip1) axis. J. Exp. Clin. Cancer Res. 38:50. 10.1186/s13046-019-1029-y 30717766 PMC6360696

[B149] Vincent E. E. Sergushichev A. Griss T. Gingras M. C. Samborska B. Ntimbane T.. (2015). Mitochondrial phosphoenolpyruvate carboxykinase regulates metabolic adaptation and enables glucose-independent tumor growth. Mol. Cell 60, 195–207. 10.1016/j.molcel.2015.08.013 26474064

[B150] Wang B. Hsu S. H. Frankel W. Ghoshal K. Jacob S. T. (2012). Stat3-mediated activation of microRNA-23a suppresses gluconeogenesis in hepatocellular carcinoma by down-regulating glucose-6-phosphatase and peroxisome proliferator-activated receptor gamma, coactivator 1 alpha. Hepatology 56, 186–197. 10.1002/hep.25632 22318941 PMC3355233

[B151] Wang P. Wu J. Ma S. Zhang L. Yao J. Hoadley K. A.. (2015). Oncometabolite D-2-hydroxyglutarate inhibits ALKBH DNA repair enzymes and sensitizes IDH mutant cells to alkylating agents. Cell Rep. 13, 2353–2361. 10.1016/j.celrep.2015.11.029 26686626 PMC4694633

[B152] Wang Y. Guo Y. R. Liu K. Yin Z. Liu R. Xia Y.. (2017). KAT2A coupled with the alpha-KGDH complex acts as a histone H3 succinyltransferase. Nature 552, 273–277. 10.1038/nature25003 29211711 PMC5841452

[B153] Wang Z. Dong C. (2019). Gluconeogenesis in cancer: function and regulation of PEPCK, FBPase, and G6Pase. Trends Cancer 5, 30–45. 10.1016/j.trecan.2018.11.003 30616754

[B154] Wei W. Liu X. Chen J. Gao S. Lu L. Zhang H.. (2017). Class I histone deacetylases are major histone decrotonylases: evidence for critical and broad function of histone crotonylation in transcription. Cell Res. 27, 898–915. 10.1038/cr.2017.68 28497810 PMC5518989

[B155] Wu S. T. Liu B. Ai Z. Z. Hong Z. C. You P. T. Wu H. Z.. (2020). Esculetin inhibits cancer cell glycolysis by binding tumor PGK2, GPD2, and GPI. Front. Pharmacol. 11:379. 10.3389/fphar.2020.00379 32292350 PMC7118906

[B156] Wu X. Miao D. Liu Z. Liu K. Zhang B. Li J.. (2020). beta-hydroxybutyrate antagonizes aortic endothelial injury by promoting generation of VEGF in diabetic rats. Tissue Cell 64:101345. 10.1016/j.tice.2020.101345 32473710

[B157] Wu Y. Zhang S. Gong X. Yu Q. Zhang Y. Luo M.. (2019). Glycolysis regulates gene expression by promoting the crosstalk between H3K4me3 and H3K14ac in *Saccharomyces cerevisiae*. J. Genet. Genomics 46, 561–574. 10.1016/j.jgg.2019.11.007 32014433

[B158] Xia Y. Yang W. Fa M. Li X. Wang Y. Jiang Y.. (2017). RNF8 mediates histone H3 ubiquitylation and promotes glycolysis and tumorigenesis. J. Exp. Med. 214, 1843–1855. 10.1084/jem.20170015 28507061 PMC5461008

[B159] Xiao M. Yang H. Xu W. Ma S. Lin H. Zhu H.. (2012). Inhibition of alpha-KG-dependent histone and DNA demethylases by fumarate and succinate that are accumulated in mutations of FH and SDH tumor suppressors. Genes Dev. 26, 1326–1338. 10.1101/gad.191056.112 22677546 PMC3387660

[B160] Xie Z. Zhang D. Chung D. Tang Z. Huang H. Dai L.. (2016). Metabolic regulation of gene expression by histone lysine beta-hydroxybutyrylation. Mol. Cell 62, 194–206. 10.1016/j.molcel.2016.03.036 27105115 PMC5540445

[B161] Xu W. Yang H. Liu Y. Yang Y. Wang P. Kim S. H.. (2011). Oncometabolite 2-hydroxyglutarate is a competitive inhibitor of alpha-ketoglutarate-dependent dioxygenases. Cancer Cell 19, 17–30. 10.1016/j.ccr.2010.12.014 21251613 PMC3229304

[B162] Yamaguchi N. Weinberg E. M. Nguyen A. Liberti M. V. Goodarzi H. Janjigian Y. Y.. (2019). PCK1 and DHODH drive colorectal cancer liver metastatic colonization and hypoxic growth by promoting nucleotide synthesis. Elife 8:e52135. 10.7554/eLife.52135.sa2 31841108 PMC7299340

[B163] Yang J. S. Hsu J. W. Park S. Y. Li J. Oldham W. M. Beznoussenko G. V.. (2018). GAPDH inhibits intracellular pathways during starvation for cellular energy homeostasis. Nature 561, 263–267. 10.1038/s41586-018-0475-6 30209366 PMC6152935

[B164] Yang W. Xia Y. Hawke D. Li X. Liang J. Xing D.. (2012). PKM2 phosphorylates histone H3 and promotes gene transcription and tumorigenesis. Cell 150, 685–696. 10.1016/j.cell.2012.07.018 22901803 PMC3431020

[B165] Yang W. Xia Y. Ji H. Zheng Y. Liang J. Huang W.. (2011). Nuclear PKM2 regulates beta-catenin transactivation upon EGFR activation. Nature 480, 118–122. 10.1038/nature10598 22056988 PMC3235705

[B166] Yen K. E. Bittinger M. A. Su S. M. Fantin V. R. (2010). Cancer-associated IDH mutations: biomarker and therapeutic opportunities. Oncogene 29, 6409–6417. 10.1038/onc.2010.444 20972461

[B167] Yi W. Clark P. M. Mason D. E. Keenan M. C. Hill C. Goddard W. A. 3rd. (2012). Phosphofructokinase 1 glycosylation regulates cell growth and metabolism. Science 337, 975–980. 10.1126/science.1222278 22923583 PMC3534962

[B168] Yin X. Choudhury M. Kang J. H. Schaefbauer K. J. Jung M. Y. Andrianifahanana M.. (2019). Hexokinase 2 couples glycolysis with the profibrotic actions of TGF-beta. Sci. Signal. 12:eaax4067. 10.1126/scisignal.aax4067 31848318

[B169] Yu Q. Tong C. Luo M. Xue X. Mei Q. Ma L.. (2017). Regulation of SESAME-mediated H3T11 phosphorylation by glycolytic enzymes and metabolites. PLoS ONE 12:e0175576. 10.1371/journal.pone.0175576 28426732 PMC5398556

[B170] Yu X. Li S. (2017). Non-metabolic functions of glycolytic enzymes in tumorigenesis. Oncogene 36, 2629–2636. 10.1038/onc.2016.410 27797379

[B171] Yu X. Ma R. Wu Y. Zhai Y. Li S. (2018). Reciprocal regulation of metabolic reprogramming and epigenetic modifications in cancer. Front. Genet. 9:394. 10.3389/fgene.2018.00394 30283496 PMC6156463

[B172] Yucel N. Wang Y. X. Mai T. Porpiglia E. Lund P. J. Markov G.. (2019). Glucose metabolism drives histone acetylation landscape transitions that dictate muscle stem cell function. Cell Rep. 27, 3939–3955 e3936. 10.1016/j.celrep.2019.05.092 31242425 PMC6788807

[B173] Zhang D. Li J. Wang F. Hu J. Wang S. Sun Y. (2014). 2-Deoxy-D-glucose targeting of glucose metabolism in cancer cells as a potential therapy. Cancer Lett. 355, 176–183. 10.1016/j.canlet.2014.09.003 25218591

[B174] Zhang D. Tang Z. Huang H. Zhou G. Cui C. Weng Y.. (2019). Metabolic regulation of gene expression by histone lactylation. Nature 574, 575–580. 31645732 10.1038/s41586-019-1678-1 PMC6818755

[B175] Zhang J. Wang J. Xing H. Li Q. Zhao Q. Li J. (2016). Down-regulation of FBP1 by ZEB1-mediated repression confers to growth and invasion in lung cancer cells. Mol. Cell. Biochem. 411, 331–340. 10.1007/s11010-015-2595-8 26546081

[B176] Zhang J. Y. Zhang F. Hong C. Q. Giuliano A. E. Cui X. J. Zhou G. J.. (2015). Critical protein GAPDH and its regulatory mechanisms in cancer cells. Cancer Biol. Med. 12, 10–22. 10.7497/j.issn.2095-3941.2014.0019 25859407 PMC4383849

[B177] Zhang L. F. Jiang S. Liu M. F. (2017). MicroRNA regulation and analytical methods in cancer cell metabolism. Cell. Mol. Life Sci. 74, 2929–2941. 10.1007/s00018-017-2508-y 28321489 PMC11107497

[B178] Zhang Q. Cai T. Xiao Z. Li D. Wan C. Cui X.. (2020). Identification of histone malonylation in the human fetal brain and implications for diabetes-induced neural tube defects. Mol. Genet. Genomic Med. 8:e1403. 10.1002/mgg3.1403 32666640 PMC7507309

[B179] Zhang S. Yu X. Zhang Y. Xue X. Yu Q. Zha Z.. (2021). Metabolic regulation of telomere silencing by SESAME complex-catalyzed H3T11 phosphorylation. Nat. Commun. 12:594. 10.1038/s41467-020-20711-1 33500413 PMC7838282

[B180] Zhang T. Guan X. W. Gribben J. G. Liu F. T. Jia L. (2019). Blockade of HMGB1 signaling pathway by ethyl pyruvate inhibits tumor growth in diffuse large B-cell lymphoma. Cell Death Dis. 10:330. 10.1038/s41419-019-1563-8 30988279 PMC6465275

[B181] Zhang X. Cao R. Niu J. Yang S. Ma H. Zhao S.. (2019). Molecular basis for hierarchical histone de-beta-hydroxybutyrylation by SIRT3. Cell Discov. 5:35. 10.1038/s41421-019-0103-0 31636949 PMC6796883

[B182] Zhang Y. Zhang X. Wang X. Gan L. Yu G. Chen Y.. (2012). Inhibition of LDH-A by lentivirus-mediated small interfering RNA suppresses intestinal-type gastric cancer tumorigenicity through the downregulation of Oct4. Cancer Lett. 321, 45–54. 10.1016/j.canlet.2012.03.013 22429998

[B183] Zhang Y. P. Liu K. L. Yang Z. Lu B. S. Qi J. C. Han Z. W.. (2019). The involvement of FBP1 in prostate cancer cell epithelial mesenchymal transition, invasion and metastasis by regulating the MAPK signaling pathway. Cell Cycle 18, 2432–2446. 10.1080/15384101.2019.1648956 31448674 PMC6739050

[B184] Zhang Z. Deng X. Liu Y. Liu Y. Sun L. Chen F. (2019). PKM2, function and expression and regulation. Cell Biosci. 9:52. 10.1186/s13578-019-0317-8 31391918 PMC6595688

[B185] Zhao D. Zou S. W. Liu Y. Zhou X. Mo Y. Wang P.. (2013). Lysine-5 acetylation negatively regulates lactate dehydrogenase A and is decreased in pancreatic cancer. Cancer Cell 23, 464–476. 10.1016/j.ccr.2013.02.005 23523103 PMC3885615

[B186] Zhao J. Li J. Fan T. W.M. Hou S. X. (2017). Glycolytic reprogramming through PCK2 regulates tumor initiation of prostate cancer cells. Oncotarget 8, 83602–83618. 10.18632/oncotarget.18787 29137367 PMC5663539

[B187] Zhao S. Lin Y. Xu W. Jiang W. Zha Z. Wang P.. (2009). Glioma-derived mutations in IDH1 dominantly inhibit IDH1 catalytic activity and induce HIF-1alpha. Science 324, 261–265. 10.1126/science.1170944 19359588 PMC3251015

[B188] Zhao W. Yang S. Chen J. Zhao J. Dong J. (2018). Forced overexpression of FBP1 inhibits proliferation and metastasis in cholangiocarcinoma cells via Wnt/beta-catenin pathway. Life Sci. 210, 224–234. 10.1016/j.lfs.2018.09.009 30193944

[B189] Zheng Q. Omans N. D. Leicher R. Osunsade A. Agustinus A. S. Finkin-Groner E.. (2019). Reversible histone glycation is associated with disease-related changes in chromatin architecture. Nat. Commun. 10:1289. 10.1038/s41467-019-09192-z 30894531 PMC6426841

[B190] Zhou L. Wang Y. Zhou M. Zhang Y. Wang P. Li X.. (2018). HOXA9 inhibits HIF-1alpha-mediated glycolysis through interacting with CRIP2 to repress cutaneous squamous cell carcinoma development. Nat. Commun. 9:1480. 10.1038/s41467-018-03914-5 29662084 PMC5902613

[B191] Zhou Y. Niu W. Luo Y. Li H. Xie Y. Wang H.. (2019). p53/Lactate dehydrogenase A axis negatively regulates aerobic glycolysis and tumor progression in breast cancer expressing wild-type p53. Cancer Sci. 110, 939–949. 10.1111/cas.13928 30618169 PMC6398928

[B192] Zhuang L. Scolyer R. A. Murali R. Mccarthy S. W. Zhang X. D. Thompson J. F.. (2010). Lactate dehydrogenase 5 expression in melanoma increases with disease progression and is associated with expression of Bcl-XL and Mcl-1, but not Bcl-2 proteins. Mod. Pathol. 23, 45–53. 10.1038/modpathol.2009.129 19838163

